# Psychiatrically relevant signatures of domain-general decision-making and metacognition in the general population

**DOI:** 10.1038/s44184-022-00009-4

**Published:** 2022-08-30

**Authors:** Christopher S. Y. Benwell, Greta Mohr, Jana Wallberg, Aya Kouadio, Robin A. A. Ince

**Affiliations:** 1 Division of Psychology, School of Humanities, Social Sciences and Law, University of Dundee, Dundee, UK; 2 School of Psychology and Neuroscience, University of Glasgow, Glasgow, UK

**Keywords:** Psychology, Human behaviour, Decision, Cognitive neuroscience, Personality

## Abstract

Human behaviours are guided by how confident we feel in our abilities. When confidence does not reflect objective performance, this can impact critical adaptive functions and impair life quality. Distorted decision-making and confidence have been associated with mental health problems. Here, utilising advances in computational and transdiagnostic psychiatry, we sought to map relationships between psychopathology and both decision-making and confidence in the general population across two online studies (*N*’s = 344 and 473, respectively). The results revealed dissociable decision-making and confidence signatures related to distinct symptom dimensions. A dimension characterised by compulsivity and intrusive thoughts was found to be associated with reduced objective accuracy but, paradoxically, increased absolute confidence, whereas a dimension characterized by anxiety and depression was associated with systematically low confidence in the absence of impairments in objective accuracy. These relationships replicated across both studies and distinct cognitive domains (perception and general knowledge), suggesting that they are reliable and domain general. Additionally, whereas Big-5 personality traits also predicted objective task performance, only symptom dimensions related to subjective confidence. Domain-general signatures of decision-making and metacognition characterise distinct psychological dispositions and psychopathology in the general population and implicate confidence as a central component of mental health.

## Introduction

When making decisions in everyday life, immediate external feedback is not always available to inform us of the utility of our choices. In the absence of external feedback, we often rely on an internally generated sense of confidence. This confidence informs metacognitive evaluations of our decisions, actions, and abilities. Though confidence and objective accuracy/utility are usually correlated, the ability to self-evaluate is often suboptimal^1^ and this can impact diverse cognitive functions such as learning, decision-making and error-monitoring^2–5^. For example, if we know that we have performed poorly on a given task, we are likely to alter our behaviour to improve future performance^6,7^. Conversely, if we lack insight, we risk persevering with damaging choices/behaviours. Indeed, deficits in metacognitive insight have been shown to contribute to impaired life quality in various neurological and psychiatric disorders^8,9^. However, the psychological determinants of metacognitive ability remain poorly understood.

Consistent relationships have been identified between metacognition and clinically relevant psychiatric symptoms, particularly general under-confidence in major depression^10–12^, under-confidence in memory in obsessive-compulsive disorder (OCD)^13–15^, and impaired metacognitive insight in schizophrenia^16–19^. However, suboptimal self-evaluation is not only restricted to clinical samples^1,20,21^. Recent studies suggest that metacognitive distortions, such as under- and over-confidence, are associated with specific personality traits^22^ and belief systems^23^ in the general population, as well as subclinical psychopathology^24–26^. For instance, symptom dimensions cutting across traditional diagnostic categories have been found to correlate with metacognitive performance in general population samples: an ‘anxious-depression’ (AD) dimension and a ‘compulsive behaviour and intrusive thought’ (CIT) dimension. Those scoring highly for CIT displayed overconfidence in perceptual decisions, reduced sensitivity of confidence judgements to objective evidence and reduced metacognitive insight^26–29^, whereas those scoring highly for AD showed low overall confidence but increased metacognitive insight^26^. These symptom-specific alterations of self-evaluation may represent enduring psychological phenotypes of psychopathology. However, metacognitive performance is governed by both domain-specific and domain-general mechanisms^30,31^ and the degree to which metacognitive abnormalities are generalisable to cognitive domains outside of perception remains unknown.

Compared to psychopathology, fewer studies have investigated relationships between metacognition and personality traits. Due to the close links between personality traits and symptoms, it is possible that personality may play a key role in relationships between metacognition and psychopathology. Overall confidence has been positively associated with extraversion^22,32^ and negatively associated with neuroticism^33^. Extraversion shows both positive and negative relationships with psychopathology^34^, negatively predicting internalizing symptoms characterised by social/interpersonal dysfunction^35^ and/or depression and anxiety^34,36^, but positively predicting externalizing symptoms characterised by exhibitionism and mania^34^. Neuroticism positively predicts many forms of psychopathology, particularly anxiety and depression^37–40^. In the current study, we sought to quantify and dissociate the degree to which dimensions of both psychopathology and personality are predictive of metacognitive performance.

We adopted a computational modelling approach to measure 1st-order decision-making and metacognition across cognitive domains. This allowed for relationships with personality and psychopathology to be grounded in quantitative model-based measures^41,42^. This is important because confidence is influenced by multiple latent processes including metacognitive sensitivity (the degree to which confidence dissociates between correct and incorrect decisions) and metacognitive bias (the absolute level of confidence experienced regardless of objective accuracy), as well as by 1st-order task performance itself^41,43^: any (or all) of which may be related to psychological dispositions. In addition to metacognitive abnormalities, some previous studies have found psychiatrically relevant 1st-order decision-making^27–29,44–46^ and/or learning^47–51^ deficits, whilst others have not^26,52,53^. Elucidation and dissociation of 1st- and 2nd-order (metacognitive) decision-making abnormalities represent key steps towards an accurate mapping of the deficits underlying core symptoms of psychopathology.

Here, across two separate online studies (*N*’s = 344 and 473, respectively), we investigated relationships between both 1st-order and metacognitive decision-making parameters, self-reported psychopathology (utilising both classic categorical and transdiagnostic approaches) and personality traits. We replicated relationships between psychiatric symptomology and decision parameters from a perceptual task across both studies. In the 2nd study we also employed a knowledge-based task to test whether the relationships are domain-general, and hence likely to have a more pervasive influence in everyday life. Finally, we investigated the degree to which personality traits influenced 1st- and 2nd-order decision-making independently of symptoms of psychopathology.

## Methods

### Participants

Participants were recruited online using the **Prolific** (www.prolific.co) and **Sona Systems** (https://www.sona-systems.com/) recruitment platforms (experiment 1: 393 participants, 16–73 years old (*M* = 25.32, *SD* = 10.83); experiment 2: 534 participants, 18–70 years old (*M* = 25.42, *SD* = 9.17)). Some participants (*N* = 374) were paid £7.50 for their time, whilst others received undergraduate course credits (*N* = 553). No *a priori* power analysis was performed for experiment 1, with the sample size being based on those employed in relevant previous studies^26,29^. However, to ensure adequate statistical power to replicate the effects observed in experiment 1, we conducted an *a priori* power analysis (using G*Power 3.1.9.7) to determine the appropriate sample size for experiment 2. We based the power analysis on the lowest significant effect size observed for a single symptom dimension across the symptom dimension-behaviour relationships in experiment 1 (Compulsive Behaviour and Intrusive Thought (CIT)-accuracy (***d’***) relationship: *f*^2^ = 0.02). The power analysis indicated that 395 participants would be required to achieve 80% statistical power to detect such an effect. Hence, the total experiment 2 sample size (534) allowed for adequate statistical power to be maintained after data exclusion.

Due to predefined exclusion criteria (explained below), 49 participants were excluded from the experiment 1 analysis, leaving a total number of 344 participants (253 female/91 male, aged from 18 to 73 years (*M* = 25.35, *SD* = 10.5)), and 61 participants were excluded from experiment 2, leaving a total number of 473 participants (233 female/240 male aged from 18 to 65 years (*M* = 25.75, *SD* = 9.24)). A *post hoc* power analysis indicated that with the final sample (473) in experiment 2, we achieved 86% statistical power to detect an effect equal to the smallest significant effect size in experiment 1 (*f*^2^ = 0.02). The only demographic information collected from participants was age and gender, thereby data anonymity was maintained. Both studies received ethical approval from the University of Dundee Research Ethics Committee and all participants provided informed consent.

### Perceptual decision task

**Fig. 1:**
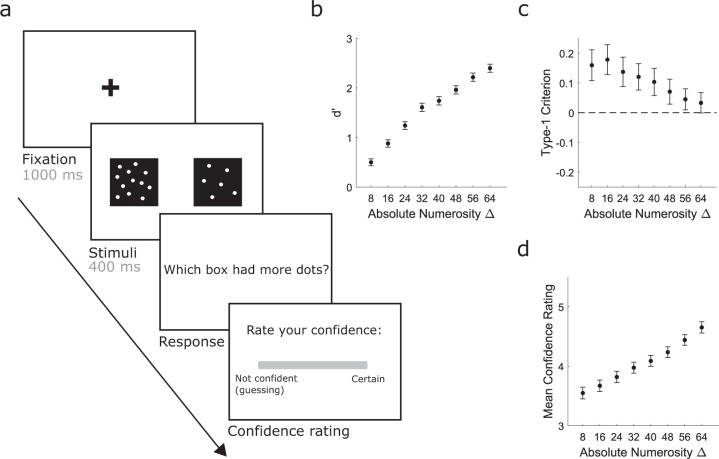
Perceptual decision-making task and behaviour in experiment 1 (*n* = 344). **a** Perceptual task. On each trial, participants judged which box (left or right) contained the higher number of dots and provided a confidence rating in each decision (scale of 1–6, where 1 represented “not confident (guessing)” and 6 represented “certain”). **b** As expected, group-averaged ***d’*** increased as a function of absolute numerosity difference. **c** Group-averaged type-1 ***c’*** were biased towards ‘left more numerous’ responses across all evidence levels and were significantly different to 0 for all numerosity differences up to 56 dots (all *p*’s < .014), but not for the easiest 64 dot difference condition (*p* = .06). This leftward bias may reflect either the pseudoneglect phenomenon, whereby neurotypical individuals tend to judge stimuli presented in the left visual field as more salient than comparable stimuli in the right visual field^75–77^, and/or a motor-response bias. **d** Group-averaged overall mean confidence ratings increased as a function of evidence strength. All error bars reflect 95% confidence intervals for the mean.

The perceptual decision task involved 2-alternative forced-choice (2-AFC) numerosity discrimination judgements with confidence ratings and was chosen to replicate Rouault et al., (2018). The perceptual decision task was employed in both experiments 1 and 2. Figure 1a shows a schematic of the trial procedure. On each trial, a black cross appeared at the centre of the screen for 1000 ms. This was followed by two black boxes, one on the left and the other on the right of the screen, which both contained numerous white dots. These were simultaneously presented for 400 ms. Participants were then asked to decide which box contained a larger number of dots by pressing the ‘w’ key for the box on the left or the ‘e’ key for the box on the right. One box (the reference box) constantly contained 272 dots (out of 544 possible dot locations), while the other box contained an increased or reduced number of dots ranging from either −72 to +72 dots (*n* = 79 in experiment 1) or −64 to +64 dots (*n* = 265 in experiment 1 and all participants in experiment 2) in increments of 8 dots in comparison to the reference box (including an identical condition). The location (left or right) of the reference box varied pseudo-randomly across trials and within each of the difficulty levels. The order of stimulus presentation was randomly generated for each participant. There was no time limit for the response and participants were not given feedback on whether their response was correct. After providing a response, participants were asked to rate how confident they were in their decision on a scale of 1 (not confident/guessing) to 6 (certain). There was no time limit for the confidence rating. Note that 82 participants in experiment 1 completed 152 trials over 2 blocks (8 trials per 19 conditions, 76 trials per block including ±72 stimuli), whereas the remaining 267 participants in experiment 1, and all participants in experiment 2, completed 136 trials over 2 blocks (8 trials per 17 conditions, 68 trials per block). Only the conditions that were shared by all participants were included in the analyses (−64 to +64 numerosity difference conditions). Participants could take a self-paced break between blocks. Before starting the task, participants completed ten practice trials in which only the easiest stimuli were presented (64 or 72 dot difference). The practice trials were identical to the experimental trials except that feedback (a green tick or red cross) was provided (indicating whether the response was correct or incorrect). Two further practice trials were used to familiarise participants with the confidence rating scale in which they were instructed how to respond if they were confident or not confident.

### Knowledge decision task

To investigate whether the psychiatric symptom – decision-making relationships generalised to other cognitive domains, in experiment 2 we employed an additional 2-AFC task which tested prior knowledge of generally known quantities: national populations^54^. Figure 3a shows a schematic of the trial procedure. On each trial, a black cross appeared at the centre of the screen for 1000 ms. This was followed by the names of two countries and the participants were required to indicate which of the two has the largest human population by selecting the corresponding button on the screen. The country names remained on the screen until the response but if the participant did not respond within 10 s, then the trial was recorded as ‘no response’. After each response, the participant was asked to rate how confident they were in their decision on a scale of 1 (not confident/guessing) to 6 (certain). No feedback about participants’ decision-making was provided during the experimental trials. There was no time limit for the confidence rating.

The national populations for creating the stimuli were downloaded from The World Bank (‘https://data.worldbank.org/indicator/SP.POP.TOTL’) in December 2019. Eight different evidence discriminability ‘bins’ were created by grouping country pairs with similar population log ratios (bins created based on log_10_ (Country A Population/Country B Population)). The log ratio bins amounted to the following, ranging from least to most discriminable: bin 1 (log_10_ ratio = 0–0.225), bin 2 = (0.225–0.45), bin 3 (0.45–0.675), bin 4 = (0.675–0.9), bin 5 (0.9–1.125), bin 6 = (1.125–1.35), bin 7 (1.35–1.575), bin 8 = (1.575–1.8). Each bin included 18 different country pairs (full task stimuli available at https://osf.io/s3cth/). The location (left or right) of the most populous country varied pseudo-randomly across trials but was counterbalanced within each of the discriminability bins (i.e., same proportion of ‘left’ larger and ‘right’ larger stimuli). The order of stimulus presentation was randomly generated for each participant. Participants completed 144 trials over 2 blocks (9 trials per 16 log ratio conditions, 72 trials per block) and could take a self-paced break between blocks. Before starting the task, 10 practice trials were completed in which only examples of the most discriminable stimuli were presented (bin 8). The practice trials were identical to the experimental trials except that feedback (a green tick or red cross) was provided (indicating whether the response was correct or incorrect).

### Modelling type-1 and type-2 sensitivity and bias

We modelled 1st-order decisions and confidence ratings on both tasks within an extended signal detection theory (SDT) framework. This model extends the classic SDT approach^55^ to quantify latent parameters (i.e., sensitivity and bias) contributing to both type-1 and type-2 decisions. Type-1 sensitivity (***d’***) indexes how accurate the participant’s 1st-order task decisions are. ***Meta-d’*** characterises type-2 (metacognitive) sensitivity as the value of ***d’*** that a metacognitively optimal observer, with the same type-1 ***criterion***, would have required to produce the observed type-2 (confidence) data. An individual with optimal metacognitive sensitivity will always be more confident when correct and less confident when incorrect. For a metacognitively ideal observer (a person who is rating confidence using the maximum possible metacognitive sensitivity), ***meta-d’*** should equal ***d’***. Importantly, we can therefore define the level of metacognitive insight/efficiency, controlling for 1st-order performance, as the value of ***meta-d’*** relative to ***d’*** (***meta-d’***/***d’***). A ***meta-d’***/***d’*** value of 1 indicates theoretically ideal metacognitive insight. A value below 1 indicates that evidence available for the type-1 decision is lost when making metacognitive judgements (type-2 decision), whereas a value above 1 indicates that more evidence is available for the type-2 decision than for the type-1 decision^41^. Note that we employed the ***meta-d’***/***d’*** measure of metacognitive efficiency, rather than the alternative ***meta-d’-d’*** measure, because it has been shown to better isolate metacognitive sensitivity from 1st-order accuracy^43^.

The confidence criteria (***type-2 c’***) represent type-2 bias calculated within the meta-d’ framework: the tendency to give high or low confidence ratings regardless of evidence strength. We calculated the absolute distance between ***type-2 c’*** and ***type-1 c’*** (|***type-2 c’*** - ***type-1 c’***|) to isolate confidence bias from perceptual response bias^56^. Lower confidence criteria (|***type-2 c’*** -***type-1 c’***|) values indicate an overall bias in favour of higher confidence ratings and higher values indicate a bias in favour of low confidence ratings (i.e., confidence criteria are inversely related to mean absolute confidence ratings). Confidence criteria values were calculated separately for each of the possible type-1 responses (i.e., ‘left’ or ‘right’ more numerous/higher population judgements in the perceptual and general knowledge tasks respectively) and for each of N-1 confidence ratings available to choose from (6 in the current experiment). To streamline the analysis, we averaged over the 5 |***type-2 c’*** - ***type-1 c’***| values for each response (‘left’ or ‘right’) separately and then averaged over the resulting ‘left’ and ‘right’ mean criteria to gain a single overall confidence criterion estimate.

**Fig. 2:**
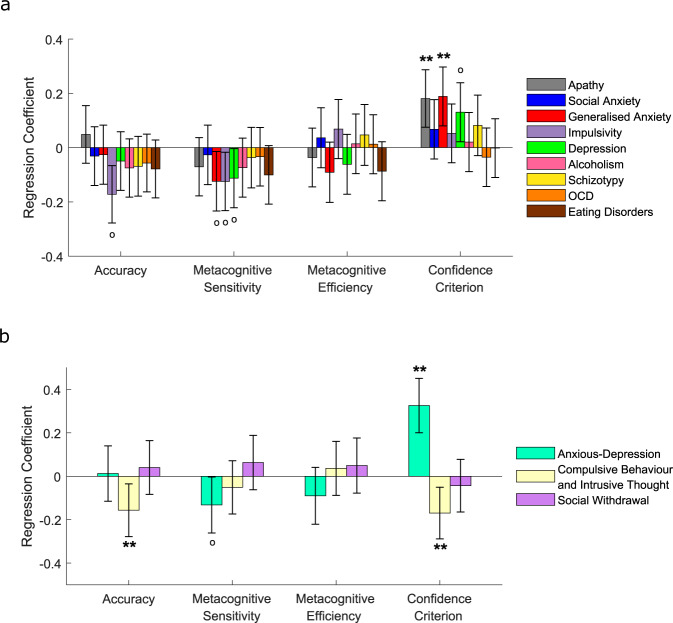
Associations between 1st- and 2nd-order decision parameters and self-reported psychopathology, additionally controlling for the influence of age and gender, in experiment 1. **a** Associations between psychiatric symptom questionnaire scores and Meta-d’ parameters from separate regression models. Given that all variables were *z*-scored prior to entry into the regression models, the *y*-axis indicates the change in each decision parameter (in standard deviations) for each change of 1 standard deviation of questionnaire scores. Accuracy = ***d'***, Metacognitive sensitivity = ***meta-d'***, Metacognitive efficiency = log(***meta-d'***/***d'***). **b** In line with previous studies^26,47^, factor analysis on the correlation matrix of all 209 questionnaire items revealed a three-factor solution comprising anxious-depression (AD), compulsive behaviour and intrusive thought (CIT) and social withdrawal (SW). The relationships between these transdiagnostic symptom dimension scores and Meta-d’ parameters were investigated using multiple regression models. CIT showed negative relationships with both 1st-order accuracy and confidence criteria, whereas AD showed a positive relationship with confidence criteria. All error bars denote 95% Confidence Intervals for the regression coefficients. °*P* < 0.05 uncorrected; **P < 0.05 Bonferroni corrected for multiple comparisons over the number of dependent variables tested.

All measures were calculated using individual participant fits (*fit_meta_d_mcmc* function) within the “Hmeta-d” toolbox^57^ (https://github.com/metacoglab/HMeta-d) in Matlab (Mathworks, USA). The input parameters for the model fits were as follows:

mcmc_params.response_conditional = 0;

mcmc_params.estimate_dprime = 0;

mcmc_params.nchains = 3;

mcmc_params.nburnin = 1000;

mcmc_params.nsamples = 10000;

mcmc_params.nthin = 1;

mcmc_params.doparallel = 0;

mcmc_params.dic = 1;

The scripts for running the fits can be found at https://osf.io/s3cth/. It is important to note that the model-based measures were calculated collapsed across all discriminability levels from each participant independently (NOT within a hierarchical model) for regressions with self-reported psychiatric symptoms and personality traits (Figs. 2, 6 & 7). However, to test the reliability of the symptom-metacognitive efficiency relationships, we also employed alternative hierarchical analysis approaches which incorporated group-level prior densities when estimating metacognitive efficiency^57,58^ (see ‘Statistical Analyses’ section below and Supplementary Figs. 7 and 8).

### Self-report psychometric questionnaires

Each participant in both experiments completed a battery of nine mental health questionnaires which assessed symptomology across a range of disorders. Symptoms of depression were measured using the Zung Self-Rating Depression Scale^59^. Obsessive-Compulsive symptoms were measured using the Obsessive-Compulsive Inventory-Revised^60^. Trait anxiety was measured using the State-Trait Anxiety Inventory Form Y-2^61^. Alcohol addiction was measured using the Alcohol Use Disorder Identification Test (AUDIT)^62^. Apathy was measured using the Apathy Evaluation Scale^63^. Eating disorder symptomology was measured using the Eating Attitudes Test (EAT-26)^64^. Impulsivity was measured using the Barratt Impulsivity Scale (BIS-11)^65^. Schizotypy was measured using the Short Scales for Measuring Schizotypy^66^. Social anxiety was measured using the Liebowitz Social Anxiety Scale which contains 24-items^67^. These questionnaires were chosen to allow us to investigate the three underlying transdiagnostic symptom dimensions identified by^47^ and replicated by^31^. In addition to the psychiatric symptom questionnaires, participants in experiment 2 also completed the Big Five Inventory^68^.

### Transdiagnostic symptom dimensions

Using the same psychiatric symptom questionnaires, Gillan et al., (2016)^47^ conducted an exploratory factor analysis (FA) on data collected in a large sample (*n* = 1413). They found that the items from all 9 mental health questionnaires (*n* = 209 items) clustered around three latent ‘factors’ which they termed ‘Anxious-Depression’, ‘Compulsive Behaviour and Intrusive Thoughts’ and ‘Social Withdrawal’ based on the individual items loading most strongly on each respective factor. The ‘Anxious-Depression’ factor was most heavily weighted by items from the Generalised Anxiety, Depression, Apathy, and Impulsivity questionnaires (see Gillan et al., 2016). The ‘Compulsive Behavior and Intrusive Thought’ factor was most heavily weighted by items from the OCD, Eating Disorders, Alcoholism and Schizotypy questionnaires. Lastly, the ‘Social Withdrawal’ factor had the highest average loadings from the Social Anxiety questionnaire. These factors have subsequently been replicated in an independent sample^26^. We replicated the FA performed by Rouault et al., (2018)^26^ to test whether the previously observed three transdiagnostic symptom dimensions^26,47^ were replicated in our data (*N* = 817 participants from both experiments 1 and 2). The analysis was conducted on the 209 individual questionnaire items using the *fa()* function from the Psych package in R, with an oblique (oblimin) rotation and maximum likelihood estimation. For the Liebowitz Social Anxiety Scale (LSAS), the average of the avoidance and fear/anxiety answers of each item was taken. In line with previous studies^26,47^, a 3-factor latent structure was found to provide the most parsimonious explanation for the item-level responses. Supplementary Fig. 3A plots correlations between the item weights from the FA performed on the current data and those of Gillan et al., (2016)^47^ for each factor. Supplementary Fig. 3B plots correlations between the individual participant scores calculated using the item weights from our FA and those of Gillan et al., (2016) for each factor.

Due to the larger sample size used to conduct their factor analysis, we applied the weights from Gillan et al., (2016) to derive scores for the three symptom dimensions for the main analyses. First, the raw responses for each item were *z*-scored across participants, the individual item z-scores within each participant were then multiplied by their corresponding factor weights and the resulting products were summed across all items for each factor. Finally, the factor sums were *z*-scored across participants in preparation for statistical analyses. Note that the results were also reproduced using the item weights from the FA performed on the current data. The R script for running the FA can be found at https://osf.io/s3cth/.

### Procedure

Both experiments were conducted online via the Gorilla experiment platform^69^. The experiments could only be completed on either a laptop, tablet, or personal computer (and not on a mobile phone) to facilitate a more optimal screen size for the visual perception task. After clicking an online link and providing informed consent, participants were first asked to provide demographic information of age and gender assigned at birth. The participants then completed the questionnaires and task(s) in a randomised order. The entire experimental session took between 40 min and 1 h for both experiments.

### Exclusion criteria

Several predefined exclusion criteria were applied to the data from both experiments to ensure acceptable data quality. Across both studies, ~23% of participants were excluded based on the criteria, leaving 344 participants for experiment 1 and 473 participants for experiment 2.

Participants who met any one or more of the following criteria in experiment 1 were excluded from all analyses:

1. Did not provide gender information (*n* = 5, 1.28%).
2. Below- or near-chance perceptual decision task performance (overall accuracy < 55%) (*n* = 9, 2.29%).
3. Below the age of 18 (*n* = 11, 2.8%).
4. Incorrect response to a ‘catch’ item employed as an attention check (*n* = 12, 3.05%). The ‘catch’ item was embedded within the Zung Depression Scale and read as follows: “If you are paying attention, please select ‘Good part of the time’ for this answer”.
5. Used the same single confidence rating across all trials of the perceptual decision task (*n* = 1, 0.25%).
6. A metacognitive efficiency (***meta-d’/d’***) ratio below 0 on the perceptual decision task (*n* = 13, 3.31%). A negative metacognitive efficiency score can occur when type-1 accuracy is around chance level and/or the participant is not using the confidence scale as expected (i.e. repeating a single confidence rating on the vast majority of trials or randomly selecting confidence ratings^70^).

Based on these criteria, a total of 49 participants (12.5%) were excluded from experiment 1.

The exclusion criteria for experiment 2 included all of those employed in experiment 1 plus additional criteria based on knowledge task performance. Again, any participants who met any one or more of the following criteria were excluded from all analyses:

1. Did not provide gender information (*n* = 0, 0%).
2. Below- or near-chance perceptual decision task performance (overall accuracy < 55%) (*n* = 19, 3.56%).
3. Below the age of 18 (*n* = 1, 0.19%).
4. Incorrect response to the ‘catch’ item employed as an attention check (*n* = 13, 2.43%).
5. Used the same single confidence rating across all trials of the perceptual decision task (*n* = 2, 0.37%).
6. A metacognitive efficiency (***meta-d’/d’***) ratio below 0 on the perceptual decision task (*n* = 27, 5.06%).
7. Below- or near-chance knowledge decision task performance (overall accuracy < 55%) (*n* = 11, 2.06%).
8. Used the same single confidence rating across all trials of the knowledge decision task (*n* = 0, 0%).
9. A metacognitive efficiency (***meta-d'/d'***) ratio below 0 on the knowledge decision task (*n* = 7, 1.31%).
10. Failed to respond on >4 trials (out of 144) on the knowledge task (*n* = 12, 2.25%).

Based on these criteria, a total of 61 participants (11.42%) were excluded from experiment 2.

### Statistical analyses

To examine the relationships between task measures and both self-reported symptoms and personality traits, we conducted a series of multiple linear regressions (always controlling for age and gender). All regressions were conducted using the *fitlm* function in MATLAB R2021a (Mathworks, USA). All variables were z-scored to ensure comparability of the regression coefficients.

The dependent measures derived from the perceptual decision-making task and the general knowledge task were type-1 accuracy (***d'***), metacognitive sensitivity (***meta-d'***), metacognitive efficiency (log(***meta-d'/d'***)) and confidence criteria (|***type-2 c*'**−***type-1 c'***|). Due to high correlations between some of the different psychiatric symptom questionnaires, we assessed relationships between individual questionnaire scores (log-transformed) and the task measures, and between individual questionnaire scores (log-transformed) and personality dimensions, in separate regression models. In the syntax of the *fitlm* function, the regressions were as follows:

$${\rm{Dependent}} \, {\rm{variable}} \sim {\log}({\rm{Questionnaire}}\, {\rm{Score}}) + {\rm{age}} + {\rm{gender}}.$$

For the regressions assessing relationships between the psychiatric symptom dimensions and the task measures, and between symptom dimensions and personality dimensions, all symptom dimensions were entered in the same regression model:

$${\rm{Dependent}}\, {\rm{variable}} \sim {\rm{Factor}}1 \, \lq{\rm{anxious}}\hbox{-}{\rm{depression}}\rq + \,{\rm{Factor}}2 \, \lq{\rm{compulsive}}\, {\rm{behaviour}}\, {\rm{and}} \,{\rm{intrusive}}\, {\rm{thought}}\rq + \,{\rm{Factor}}3 \, \lq{\rm{social}}\, {\rm{withdrawal}}\rq + {\rm{age}} + {\rm{gender}}.$$

This was also the case for the regressions assessing relationships between personality dimensions and task measures, whilst controlling for symptom dimensions:

$${\rm{Dependent}}\, {\rm{variable}} \sim {\rm{extraversion}} + {\rm{agreeableness}} + \,{\rm{conscientiousness}} + \,{\rm{openness}}\, + \,{\rm{neuroticism}} + \,{\rm{Factor}}1 \, \lq{\rm{anxious}}\hbox{-}{\rm{depression}}\rq + \,{\rm{Factor}}2 \, \lq{\rm{compulsive}}\, {\rm{behaviour}}\, {\rm{and}}\,{\rm{intrusive}}\, {\rm{thought}}\rq + \,{\rm{Factor}}3\, \lq{\rm{social}}\, {\rm{withdrawal}}\rq + {\rm{age}} + {\rm{gender}}.$$

To correct for multiple comparisons, Bonferroni correction was applied over the number of dependent variables tested in each different analysis. For the individual questionnaire-behaviour relationships presented in Figs. 2a and 6a, c and Supplementary Fig. 6A, the corrected alpha level was 0.0014. For the symptom dimension-behaviour relationships presented in Figs. 2b and 6b, d and Supplementary Fig. 6B, the corrected alpha level was 0.0125. For the personality dimension-behaviour relationships presented in Fig. 7a, b, the corrected alpha level was 0.0167. For the individual questionnaire-personality relationships presented in Fig. 8a, the corrected alpha level was 0.0011. For the symptom dimension-personality relationships presented in Fig. 8b, the corrected alpha level was 0.01.

Pearson correlation coefficients were calculated for each of the between-subject correlations of interest. Paired- and independent-samples *t*-tests were employed to test for differences in decision parameters both within- and between-tasks.

For analysis of the relationships between psychiatric symptom dimensions and metacognitive efficiency, in addition to the linear regression approach outlined above, we also adopted two approaches which have recently been employed to test for group differences and to link external qualities to metacognitive efficiency^57,58^. These approaches incorporate Bayesian priors to constrain estimates of both group-average and individual participant metacognitive efficiency using hierarchical modelling. Two separate analyses were performed using the hierarchical fitting option in the “HMeta-d” toolbox^57^. These analyses were conducted to test the reliability of the null relationships between psychiatric symptom dimensions and metacognitive efficiency observed in the multiple linear regression analyses performed using non-hierarchical individual participant Meta-d’ fits (presented in Figs. 2, 6 and 7). For both hierarchical analyses, we used the perception task data from both experiments combined (*N* = 817) to maximize statistical power.

In the first hierarchical analysis, we used median splits to create ‘high’ and ‘low’ symptom dimension score groups for each of the three dimensions (AD, CIT, and SW). The hierarchical Bayesian estimation implemented in HMeta-d’ specifies group-level prior densities over each of the participant-level parameters and provides a group-level estimate of metacognitive efficiency (***meta-d’/d’***). We estimated group-level metacognitive efficiency separately for the high and low symptom groups across all three symptom dimensions. The group-level fits were performed using the *fit_meta_d_mcmc_group* function^57^ with the following input parameters:

mcmc_params.response_conditional = 0;

mcmc_params.estimate_dprime = 0;

mcmc_params.nchains = 3;

mcmc_params.nburnin = 1000;

mcmc_params.nsamples = 10000;

mcmc_params.nthin = 1;

mcmc_params.doparallel = 0;

mcmc_params.dic = 1;

Group difference in metacognitive efficiency were assessed by first calculating the distribution of differences in posterior parameter samples from each group (high > low), and then determining the 95% highest-density interval (HDI) for this distribution. The group-level posterior densities were then used to test the statistical significance of differences in metacognitive efficiency. Specifically, if the 95% highest-density interval (HDI) of the difference between groups did not include 0 then the difference was judged to be statistically significant, whereas if the HDI did include 0 then the difference was judged not statistically significant^57^.

In the second hierarchical analysis, we adopted a recently developed approach which allows for relationships between potential covariates and metacognitive efficiency (***meta-d'/d'***) to be estimated within the hierarchical meta-d’ model^57,58^. This approach embeds the estimation of symptom-metacognitive efficiency relationships into the parameter inference routine, such that the group-level estimate of regression coefficients reflects the influence of individual differences in symptom severity on metacognitive efficiency^58^. The regressors included in the hierarchical model were Age, Gender, AD scores, CIT scores and SW scores, with the outcome variable being metacognitive efficiency (***meta-d'/d'***) scores. The fitting was performed using the *fit_meta_d_mcmc_regression* function^57^ with the following input parameters:

mcmc_params.response_conditional = 0;

mcmc_params.estimate_dprime = 0;

mcmc_params.nchains = 3;

mcmc_params.nburnin = 1000;

mcmc_params.nsamples = 10000;

mcmc_params.nthin = 1;

mcmc_params.doparallel = 0;

mcmc_params.dic = 1;

Again, posterior densities were used to test the statistical significance of the regression coefficients. Specifically, if the 95% highest-density interval (HDI) of a regression coefficient did not include 0 then the relationship was judged to be statistically significant, whereas if the HDI did include 0 then the relationship was judged not statistically significant.

## Results

In study 1 (*N* = 344 after data exclusion), participants performed a visual two-alternative forced-choice (2-AFC) numerosity discrimination task (Fig. 1a) and completed a battery of nine self-report psychiatric symptom questionnaires. The task involved deciding which of two simultaneously presented black boxes contained a greater number of white dots on each trial, and then rating confidence in the decision (on a scale of 1—‘not confident (guessing)’ to 6—‘certain’). The true numerosity difference between the boxes (and hence task difficulty) was manipulated from trial-to-trial. Figure 1 provides a schematic of the trial procedure and an overview of task performance.

### Psychiatric symptom dimensions are associated with dissociable 1st-order and metacognitive decision-making signatures

To quantify latent parameters contributing to both 1st- and 2nd-order decisions, we modelled the task data within an extended signal detection theory (SDT) framework^41,42,57,71^. This provided measures of both 1st-order accuracy (***d’***) and the degree to which confidence ratings dissociated correct from incorrect decisions (metacognitive sensitivity (***meta-d′***)) (see Methods for full details). Because ***d'*** and ***meta-d'*** are measured in the same units (signal-to-noise ratio), their ratio can be used to index the level of metacognitive efficiency of the observer^43,57^. This measure quantifies how much of the information available for 1st-order decisions is retained when rating confidence. The *meta-d*' model also separates sensitivity measures from measures of both 1st-order (***criterion (c')***) and 2nd-order (***confidence criteria***) bias.

We investigated relationships between self-reported psychiatric symptoms and the task measures of interest (perceptual accuracy (***d'***), metacognitive sensitivity (***meta-d'***), metacognitive efficiency (***meta-d'/d'***), confidence criteria), whilst controlling for age and gender (see Methods). Note that to calculate the task measures, individual meta-d’ fits were applied to the data (collapsed across all levels of absolute numerosity difference) from each participant independently (NOT within a hierarchical model), thereby providing overall metrics of both perceptual and metacognitive performance for each participant which were independent of the data from other participants. This importantly satisfies the assumption that observations should be independent of each other for regression analysis. Full sample distributions of all measures are shown in Supplementary Fig. 1, and relationships with demographic variables (age and gender) are reported in Supplementary Fig. 2 and Supplementary Results.

Figure 2a plots standardised regression coefficients indexing the strength and direction of the relationships between questionnaire scores and each task measure. Self-reported apathy (*β* = 0.18, *p* = .033, corrected) and generalised anxiety (*β* = 0.19, *p* = .027, corrected) were positively associated with confidence criteria (indicating negative relationships with absolute confidence). No other relationships survived Bonferroni correction.

As well as relating scores on each questionnaire separately, we performed a transdiagnostic analysis^26,47^ to relate underlying dimensions of psychopathology to both perceptual and metacognitive performance. The transdiagnostic approach accounts for high comorbidity between diagnostic categories (indicated by strong correlations between individual questionnaire scores (Supplementary Fig. 2A)) and potentially heterogenous symptom clusters within categories^72,73^. The questionnaires were chosen to match those of previous studies^26,47^ that used factor analysis to identify three symptom dimensions underlying the 209 items across all nine questionnaires: an ‘anxious-depression’ (AD) dimension, a ‘compulsive behaviour and intrusive thought’ (CIT) dimension and a ‘social withdrawal’ (SW) dimension. We conducted the same factor analysis in our entire sample (across both studies: *N* = 817) and replicated the three dimensions (Supplementary Fig. 3).

We tested relationships between the symptom dimensions and task measures, again controlling for age and gender (Fig. 2b). The CIT dimension showed a dissociation between 1st- and 2nd-order effects: despite being associated with lower objective accuracy (*β* = −0.16, *p* = .047, corrected), CIT was also associated with reduced confidence criteria (indicating high levels of absolute confidence) (*β* = −0.17, *p* = .022, corrected). Conversely, whilst the AD dimension showed no relationship with objective accuracy (*β* = 0.01, *p* = .85), it was associated with increased confidence criteria (indicating low levels of absolute confidence) (*β* = 0.33, *p* < .001, corrected). The confidence criteria effects replicate Rouault, Seow, et al. (2018)^26^ who found AD/CIT to be associated with low/high levels of absolute confidence, respectively. It is noteworthy that the CIT-confidence effect was not captured in the standard questionnaire analyses (Fig. 2a), and therefore the transdiagnostic approach revealed relationships masked by classic diagnostic categories.

Overall, the results of study 1 show that dissociable psychiatric symptom dimensions are associated with distinct 1st-order and metacognitive decision-making signatures, with CIT predicting reduced perceptual accuracy but high absolute confidence levels and AD predicting low absolute confidence levels despite intact perceptual accuracy.

### Both domain-specific and domain-general factors contributed to performance, and confidence was the most strongly correlated measure across cognitive domains

**Fig. 3:**
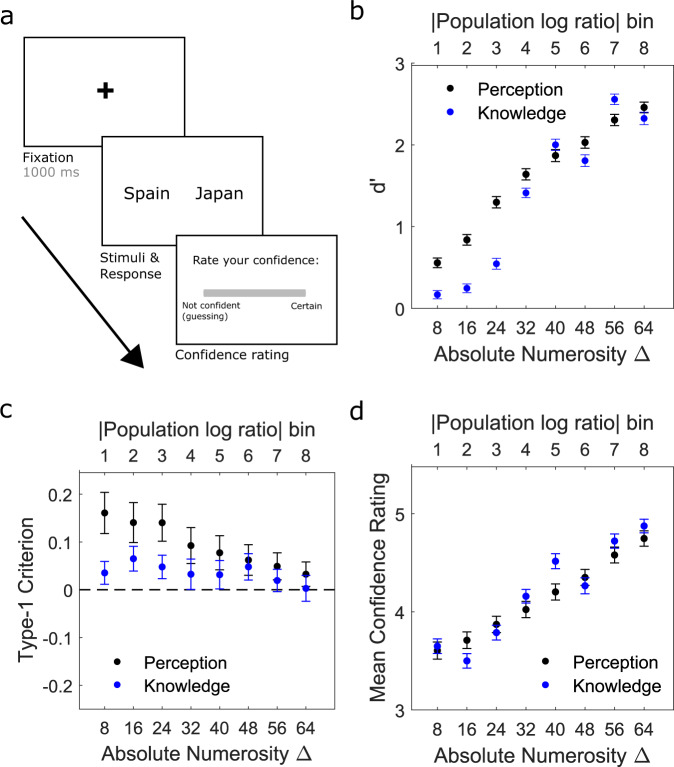
Knowledge decision-making task and behaviour in study 2 (*n* = 473). **a** In addition to the perception task, participants also completed a task which tested knowledge of national populations. On each trial, participants judged which of two countries had the higher human population and provided a confidence rating (scale of 1–6, where 1 represented “not confident (guessing)” and 6 represented “certain”). Eight evidence discriminability bins were created by grouping pairs of countries with similar population log ratios. The log ratio bins amounted to the following, ranging from least to most discriminable: bin 1 (log10 ratio = 0–0.225), bin 2 = (0.225–0.45), bin 3 (0.45–0.675), bin 4 = (0.675–0.9), bin 5 (0.9–1.125), bin 6 = (1.125–1.35), bin 7 (1.35–1.575), bin 8 = (1.575–1.8). **b** In both tasks, group-averaged ***d’*** increased as a function of evidence strength**. c** The systematic type-1 leftward biases (here indexed by the mean type-1 ***c’***) decreased as a function of evidence level for both tasks but were systematically stronger for the perceptual task. **d** Group-averaged overall mean confidence ratings increased as a function of evidence strength. All error bars reflect 95% confidence intervals for the mean.

In a 2nd study (*N* = 473 after data exclusion), we sought to extend the results in an independent sample by testing (1) whether the relationships generalise across cognitive domains and (2) whether big-5 personality dimensions explain additional variance in either 1st- and/or 2nd-order decision measures, over and above that explained by symptom dimensions. Participants performed the same perceptual task but also performed an additional 2-AFC task which tested prior knowledge of generally known quantities: national populations (Fig. 3a)^54,74^. The knowledge task was chosen to maintain a similar trial and response structure to the perceptual task while indexing performance in a different cognitive domain. The task involved judging which of two countries had the highest human population, and then rating decision confidence on the same 6-point scale (1—‘not confident (guessing)’ to 6—‘certain’). The true population difference between the two countries (and hence task difficulty) was manipulated from trial-to-trial (Methods). Figure 3 provides a schematic of the trial procedure and an overview of performance on both tasks. In addition to the nine psychiatric symptom questionnaires, participants also completed the Big Five Inventory (BFI)^68^ to assess personality dimensions of ‘extraversion’, ‘agreeableness’, ‘conscientiousness’, ‘openness to experience’ and ‘neuroticism’. Full sample distributions of outcome measures for study 2 are shown in Supplementary Fig. 4 and relationships with age and gender are reported in Supplementary Fig. 5 and Supplementary Results.

**Fig. 4:**
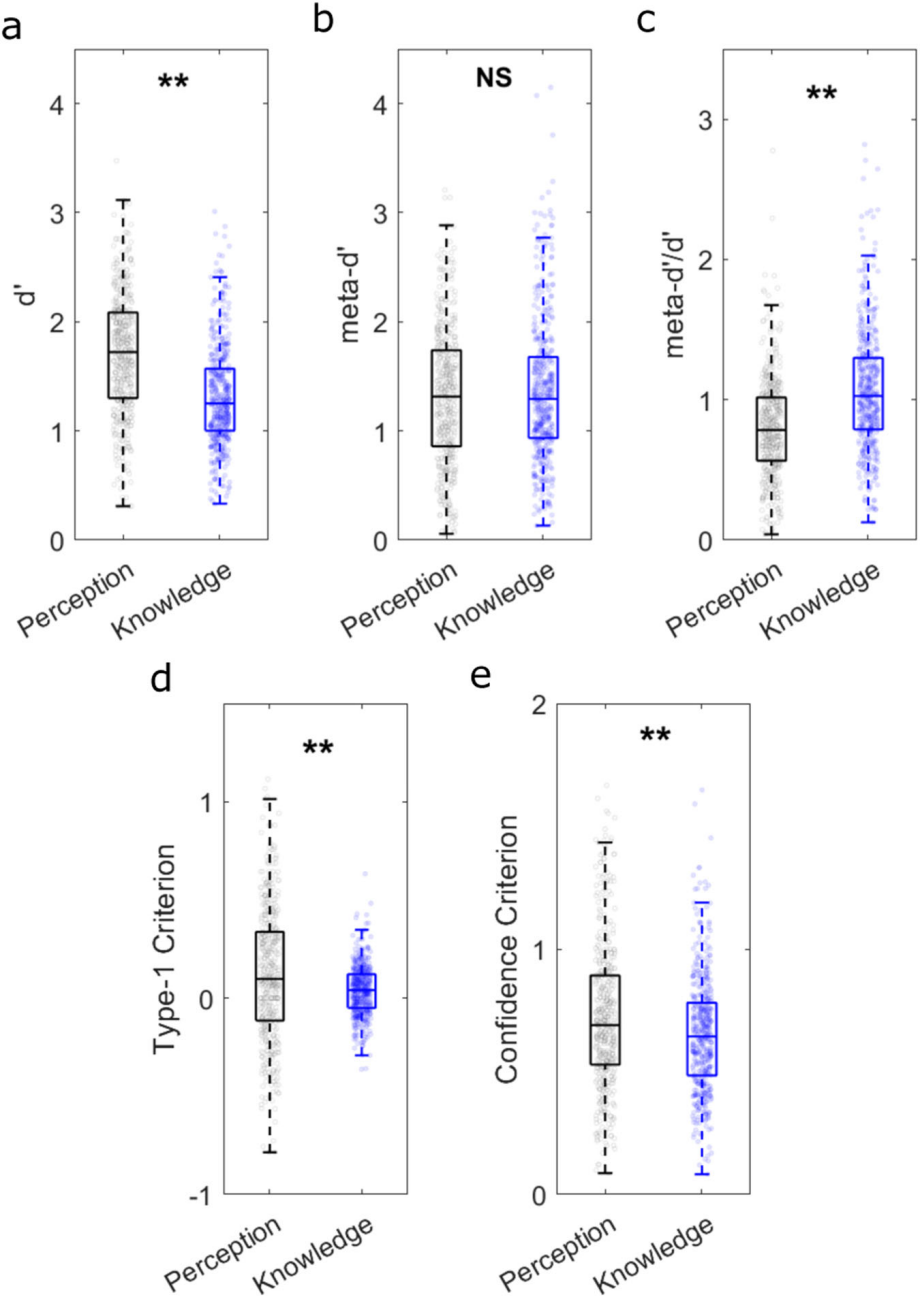
Between-task comparisons of overall performance. The data are shown for (**a**) type-1 accuracy (***d'***), (**b**) metacognitive sensitivity (***meta-d’***), (**c**) metacognitive efficiency (***meta-d’***/***d’***), (**d**) criterion (***type-1 c’***) and (**e**) type-2 criterion (***confidence c'***). On each box, the central line is the median, the edges of the box are the 25th and 75th percentiles, and the whiskers extend ±2.7 standard deviations from the median. *P < 0.05, **P < 0.01.

Comparing performance between the tasks (Fig. 4), participants performed better on the perceptual (mean ***d’*** = 1.70; SD = 0.56) compared to the knowledge (mean ***d'*** = 1.32; SD = 0.47) task (*t*(472) = 13.25, *p* < .001). However, ***meta-d’*** did not significantly differ (mean perceptual ***meta-d'*** = 1.32; SD = 0.63, mean knowledge ***meta-d'*** = 1.37; SD = 0.66: *t*(472) = −1.32, *p* = .186). ***Meta-d'*** values were more closely aligned with ***d'*** values in the knowledge task, as can be seen by comparing Fig. 4a, b. Accordingly, overall metacognitive efficiency was higher for knowledge (mean ***meta-d'***/***d'*** = 1.07; SD = 0.44) relative to perception (mean ***meta-d'***/***d'*** = 0.8; SD = 0.36) (*t*(472) = 10.9, *p* < .001) (Fig. 4c). Leftward group-level response biases (indexed by ***c’***) were significantly stronger for perception (mean perceptual ***c'*** = 0.12; SD = 0.34, mean knowledge ***c'*** = 0.04; SD = 0.13: *t*(472) = 5.01, *p* < .001) (Fig. 4d). Given that the leftward bias was present for both tasks, but stronger for perception, suggests that both motor and perceptual biases likely contributed^75–77^. Finally, despite the knowledge task being objectively more difficult than the perceptual task (as reflected by the ***d’*** differences), knowledge ***confidence criteria*** were lower (indicating higher mean confidence ratings) (mean perceptual ***confidence c'*** = 0.73; SD = 0.3, mean knowledge ***confidence c'*** = 0.65; SD = 0.24: *t*(472) = −5.93, *p* < .001) (Fig. 4e).

**Fig. 5:**
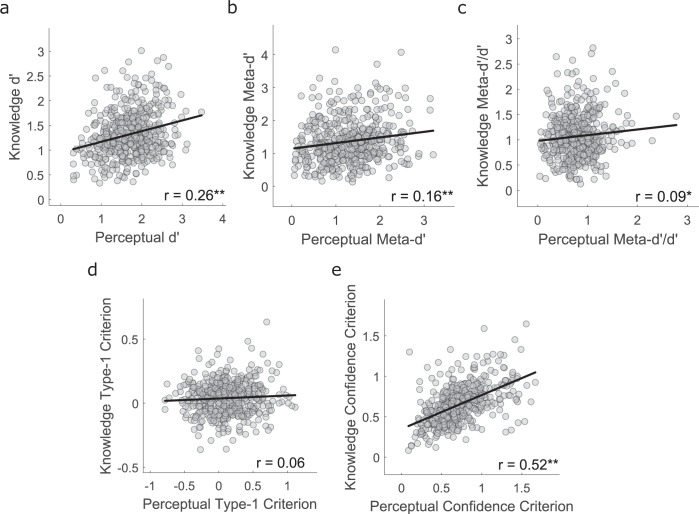
Between-participant Pearson correlations across the two tasks. Data are plotted for overall (**a**) type-1 accuracy (***d'***), (**b**) metacognitive sensitivity (***meta-d'***), (**c**) metacognitive efficiency (***meta-d'***/***d'***), (**d**) criterion (***type-1 c'***) and (**e**) type-2 criterion (***confidence c'***). *P < 0.05, **P < 0.01.

To estimate the contribution of domain-general mechanisms, we tested the correlation of each measure (collapsed across evidence levels) between tasks (Fig. 5). We reasoned that significant correlation of a given measure between tasks suggests that a shared latent mechanism must contribute across cognitive domains^78,79^. The only non-significant correlation was for ***c'*** (*r*(471) = 0.06, *p* = .18). All other correlations indicated influence of domain-general mechanisms on performance, though with marked differences in correlation strength across measures. Both ***d'*** (*r*(471) = 0.26, *p* < .001) and ***meta-d'*** (*r*(471) = 0.16, *p* < .001) showed moderate correlations across tasks, whilst ***meta-d'/d'*** (*r*(471) = 0.09, *p* = .043) showed the weakest correlation of the significant measures. In line with previous studies^78,79^, the most strongly correlated measure across tasks was ***confidence c'*** (*r*(471) = 0.52, *p* < .001), suggesting that overall confidence calibration represents a stable, ‘trait-like’ measure which strongly influences metacognitive judgements across cognitive domains. It is important to note that estimates of ***confidence c'*** may be inherently less noisy than estimates of ***meta-d'*** and ***meta-d'/d'***, and that this may contribute to the differences in correlation strength of these measures across tasks. Further work is needed to ascertain whether absolute confidence levels are indeed an inherently more stable trait across cognitive domains than metacognitive sensitivity/efficiency.

### Psychiatrically relevant 1st- and 2nd-order decision-making signatures are domain-general

**Fig. 6:**
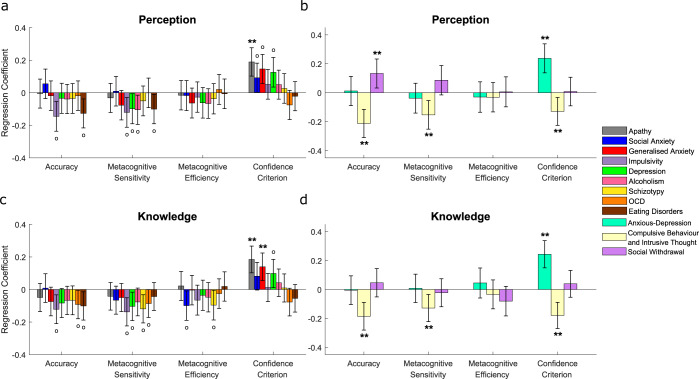
Associations between 1st- and 2nd-order decision parameters and self-reported psychopathology, additionally controlling for age and gender, in experiment 2. **a** Associations between psychiatric symptom questionnaire scores and *perceptual* Meta-d’ parameters. Given that all variables were z-scored prior to entry into the regression models, the *y*-axis indicates the change in each decision parameter (in standard deviations) for each change of 1 standard deviation of questionnaire scores. Accuracy = ***d'***, Metacognitive sensitivity = ***meta-d'***, Metacognitive efficiency = log(***meta-d'***/***d'***). **b** Associations between transdiagnostic symptom dimension scores and *perceptual* Meta-d' parameters. **c** Associations between psychiatric symptom questionnaire scores and *knowledge* Meta-d’ parameters. **d** Associations between transdiagnostic symptom dimension scores and *knowledge* Meta-d' parameters. All error bars denote 95% Confidence Intervals for the regression coefficients. °*P* < 0.05 uncorrected; **P < 0.05 corrected for multiple comparisons over the number of dependent variables tested.

Next, we investigated whether the relationships between psychiatric symptoms and task measures are themselves domain-specific or domain-general. For perception, similar relationships between task measures and both individual questionnaires and symptom dimensions were observed to those in experiment 1, though in experiment 2 additional significant relationships were found between CIT and metacognitive sensitivity (*β* = −0.15, *p* = .013, corrected) and between SW and 1st-order accuracy (*β* = 0.13, *p* = .048, corrected) (see Fig. 6a, b compared to Fig. 2a, b). The perception-symptom relationships across both studies combined (*N* = 817) are presented in Supplementary Fig. 6. To test whether the relationships generalised across cognitive domains, we turned to the knowledge task (Fig. 6c, d). In line with perception, knowledge confidence criteria were positively associated with apathy (*β* = 0.18, *p* < .001, corrected) and generalised anxiety (*β* = 0.14, *p* = .047, corrected).

The knowledge task-symptom dimension results closely replicated those of the perceptual task (Fig. 6d). CIT was associated with reduced 1st-order accuracy (*β* = −0.19, *p* < .001, corrected) and metacognitive sensitivity (*β* = −0.13, *p* = .039, corrected) as well as reduced confidence criteria (*β* = −0.18, *p* < .001, corrected), whereas AD was positively associated with confidence criteria (*β* = 0.24, *p* < .001, corrected). However, SW and 1st-order accuracy were not correlated for the knowledge task (*β* = 0.05, *p* = .351). As in experiment 1, no significant relationships with metacognitive efficiency were found for any of the symptom dimensions in either task. Importantly, these null results held when we further tested them (on the combined perceptual data from both experiments) using alternative hierarchical analysis approaches which incorporated group-level prior densities when estimating metacognitive efficiency^57,58^ (see Supplementary Results and Supplementary Figs. 7 and 8). Hence, we found no evidence for any relationship between symptom dimensions and metacognitive efficiency. Note that the negative relationships between CIT and metacognitive sensitivity in both tasks may be accounted for by the relationships between CIT and 1st-order accuracy, given that ***meta-d'*** positively correlates with ***d'***. Indeed, the lack of any relationship between CIT and metacognitive efficiency (***meta-d'***/***d'***) indicates that CIT is primarily associated with 1st-order accuracy rather than metacognitive sensitivity. Overall, overlap in relationships with psychiatric symptoms between the perceptual and knowledge tasks suggests that domain-general mechanisms largely underlie the associations between distinct dimensions of psychopathology and 1st-order and metacognitive decision signatures.

### Personality explains additional variance in 1st-order decisions, but not confidence

Having established domain-general associations with dimensions of psychopathology, we next investigated whether Big-5 personality traits account for additional variance in 1st- and/or 2nd-order performance across both tasks.

**Fig. 7:**
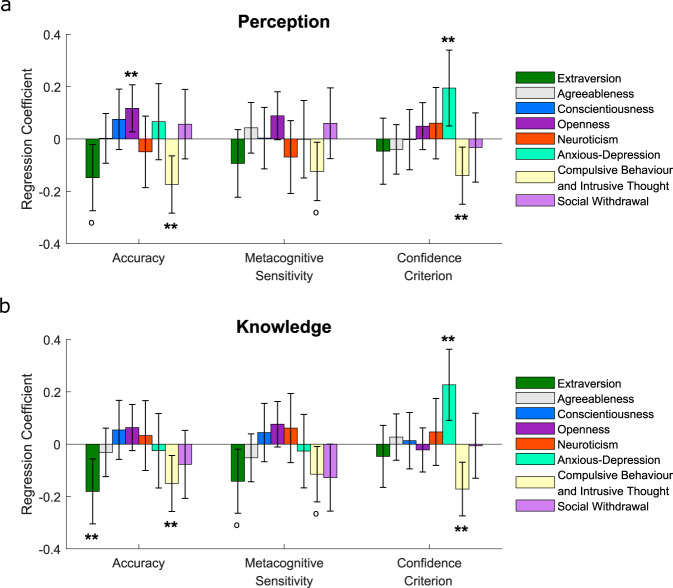
Associations between 1st- and 2nd-order decision parameters and both self-reported personality traits and symptom dimensions, controlling for age and gender, in experiment 2. Data are plotted separately for the (**a**) perception and (**b**) knowledge tasks. Note that these analyses were only performed for ***d'***, ***meta-d’*** and ***confidence c'*** as no relationships were found with metacognitive efficiency (***meta-d'***/***d'***) for any of the symptom or personality dimensions when tested alone. All error bars denote 95% Confidence Intervals for the regression coefficients. °*P* < 0.05 uncorrected; **P < 0.05 corrected for multiple comparisons over the number of dependent variables tested.

We entered Big-5 factor scores into regression models as predictors along with the symptom dimensions (and age and gender) (Fig. 7). Note that variance inflation factors (VIFs) were ≤2.83 for all predictors, indicating a negligible influence of multicollinearity on the estimated coefficients^80^. The analysis was only performed for ***d'***, ***meta-d'*** and ***confidence c'*** as no relationships were found with metacognitive efficiency (***meta-d'***/***d'***) for any of the symptom (Fig. 6b, d) or personality (Supplementary Fig. 9) dimensions when tested independently. For the personality dimensions, extraversion was negatively correlated with 1st-order accuracy on the knowledge task (*β* = −0.18, *p* = .014, corrected) and a similar but weaker negative relationship was observed on the perceptual task (*β* = −0.15, *p* = .023, uncorrected). Additionally, openness to experience was positively correlated with 1st-order accuracy on the perception task (*β* = 0.12, *p* = .034, corrected). With personality dimensions included in the regression models, CIT scores remained significant independent predictors of 1st-order accuracy for both tasks (perception: *β* = −0.17, *p* = .006, corrected; knowledge: *β* = −0.15, *p* = .019, corrected).

No personality dimensions significantly predicted metacognitive performance (***meta-d’*** or ***confidence c’***) in either task after multiple comparison correction, whereas confidence criteria were positively related to AD (perception: *β* = 0.19, *p* = .026, corrected; knowledge: *β* = 0.23, *p* = .003, corrected), and negatively related to CIT (perception: *β* = −0.14, *p* = .036, corrected; knowledge: *β* = −0.17, *p* = .003, corrected) for both tasks. Hence, whilst both personality and psychiatric symptom dimensions were independently associated with 1st-order accuracy, symptom dimensions were the only significant predictors of domain-general confidence.

### Transdiagnostic symptom dimensions elucidate relationships between personality traits and psychopathology

**Fig. 8:**
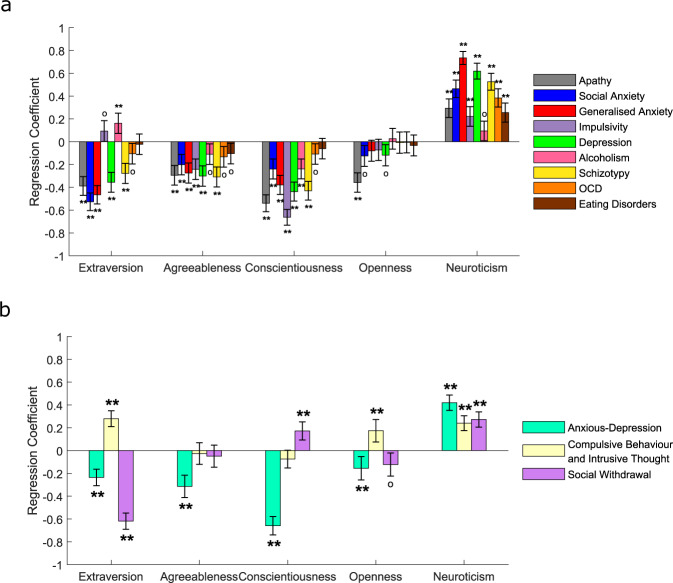
Widespread associations between self-reported personality traits and psychopathology, controlling for the influence of age and gender. **a** Associations between psychiatric symptom questionnaire scores and personality dimension scores from separate regression models. The *y*-axis indicates the change in each personality dimension score for each change of 1 standard deviation of questionnaire scores. **b** Associations between transdiagnostic symptom dimension scores and personality dimension scores. All error bars denote 95% Confidence Intervals for the regression coefficients. °*P* < 0.05 uncorrected; **P < 0.05 corrected for multiple comparisons over the number of dependent variables tested.

Finally, we investigated relationships between Big-5 personality dimensions and symptoms of psychopathology (Fig. 8). Controlling for age and gender, extraversion was negatively associated with apathy (*β* = −0.39, *p* < .001, corrected), social anxiety (*β* = −0.53, *p* < .001, corrected), generalised anxiety (*β* = −0.47, *p* < .001, corrected), depression (*β* = −0.36, *p* < .001, corrected) and schizotypy (*β* = −0.28, *p* < .001, corrected), but positively associated with alcoholism (*β* = 0.16, *p* = .017, corrected). Agreeableness was significantly negatively associated with scores on 6 out of 9 questionnaires (all *β*’s ≤ −0.1, all *p*’s ≤ .001, corrected). Conscientiousness was significantly negatively associated with scores on 7 questionnaires (all *β*’s ≤ −0.11, all *p*’s ≤ .001, corrected). Openness to experience was negatively associated with apathy (*β* = −0.36, *p* < .001, corrected). Neuroticism was significantly positively associated with scores on 8 of the questionnaires (all *β*’s ≥ 0.09, all *p*’s ≤ .001, corrected).

For symptom dimensions (Fig. 8b), extraversion was negatively associated with both AD (*β* = −0.24, *p* < .001, corrected) and SW (*β* = −0.62, *p* < .001, corrected), but positively associated with CIT (*β* = 0.28, *p* < .001, corrected). Only AD showed a significant negative association with agreeableness (*β* = −0.31, *p* < .001, corrected), suggesting that this transdiagnostic dimension may account for the ubiquitous negative relationships observed across the individual questionnaires (Fig. 8a). For conscientiousness, AD was negatively associated (*β* = −0.66, *p* < .001, corrected) whilst SW was positively associated (*β* = 0.17, *p* < .001, corrected). This suggests that AD may also account for the negative relationships between multiple questionnaires and conscientiousness (Fig. 8a). Openness was negatively correlated with AD (*β* = −0.15, *p* = .016, corrected), but positively correlated with CIT (*β* = 0.17, *p* = .002, corrected). It is notable that no positive relationships were observed between either conscientiousness or openness and any of the individual questionnaire scores (Fig. 8a), whereas the transdiagnostic analysis revealed positive relationships with SW (conscientiousness) and CIT (openness), respectively (Fig. 8b). Hence, the transdiagnostic approach revealed relationships which were masked by classic diagnostic categories. Finally, neuroticism was positively associated with all three symptom dimensions (all *β*’s ≥ 0.24, all *p*’s < .001, corrected). The results confirm strong relationships between dimensions of personality and psychopathology and highlight that the transdiagnostic approach provides information about the nature of these relationships which is not apparent using classical diagnostic categories.

## Discussion

Distortions of both 1st-order perceptual decision-making and metacognitive evaluation have been suggested to characterise various forms of psychopathology. To date it has remained unclear exactly which latent processes are involved and whether the distortions generalise across cognitive domains. Here, employing a battery of self-report psychiatric symptom questionnaires and computational modelling of psychophysical performance across two studies, we found a symptom dimension characterised by ‘compulsive behaviour and intrusive thought’ (CIT) to be associated with reduced 1st-order objective accuracy but, paradoxically, increased confidence. Conversely, an ‘anxious-depression’ (AD) dimension was associated with systematically low absolute confidence in the absence of any relationship with 1st-order accuracy. These relationships replicated across perception and general knowledge tasks and occurred independently of age and gender. Alongside dimensions of psychopathology, we also investigated whether Big-5 personality traits explained additional variance in either 1st-order and/or metacognitive decision-making. Whilst dimensions of both personality (extraversion, openness) and symptoms (CIT) were independently associated with 1st-order accuracy, only symptom dimensions (AD, CIT) predicted metacognitive performance. Overall, the results reveal robust, domain-general signatures of decision-making and metacognition related to distinct psychological dispositions and psychopathology in the general population, and further elucidate the nature of relationships between personality and psychopathology.

The CIT dimension most prominently links features of impulsivity, OCD, schizotypy, addiction and eating disorders. Our results suggest domain-general alterations across multiple levels of the decision hierarchy in CIT, in line with previous studies which have found compulsivity to be associated with alterations in 1st-order perceptual decision-making^27,28,81^, goal-directed control^9,47,51,82^ and confidence judgements^26,28,29^. The CIT dimension was associated with a positive confidence bias (across both experiments and tasks) and reduced metacognitive sensitivity (across both tasks but only in study 2) but showed no relationship with metacognitive efficiency. Previous studies have found a reduction in metacognitive efficiency associated with compulsivity^26,27^ but we did not find evidence for this here. The lack of an association with metacognitive efficiency suggests that the relationship between CIT and metacognitive sensitivity (***meta-d*'**) may have been driven by the negative relationship between CIT and first order accuracy (***d'***). Our results suggest that confidence ratings still dissociate between correct and incorrect trials to the degree expected given the 1st-order performance in CIT, but overall confidence calibration is high. The apparent contradiction of reduced objective performance but inflated confidence is in line with an altered connection between confidence and behaviour^29^.

The 1st-order decision deficits associated with CIT, and related disorders, have been attributed to alterations in decision formation processes such as evidence accumulation^27,44,83^. Here we show that the deficits extend beyond decisions about external sensory stimuli to include semantic memory/knowledge decisions based on internal evidence. Hence, they cannot be explained by low level sensory dysfunction. Higher order deficits in the internal modelling of task structures have also been shown to characterise compulsivity^9,82,84^. However, as optimal performance on our tasks did not require participants to learn underlying state transition probabilities, but rather depended in a straightforward manner on their decision accuracy on each individual trial, it seems unlikely that impaired internal task models can explain the 1st-order effects observed here. The effects may be explained by a recently proposed ‘decision acuity’ (*d*) trait found to underlie decision-making performance, independently of IQ, across a large range of decision tasks^46^. Interestingly, both *d* and IQ scores were found to be negatively related to a psychiatric dimension characterised by compulsivity/obsessionality/schizotypy (labelled ‘aberrant thinking’)^46^.

The AD dimension, which most prominently linked features of apathy, anxiety, and depression, was associated with low confidence across cognitive domains in the absence of any relationships with objective performance. These findings confirm negative confidence bias as a feature of anxious-depressive symptomology, even in sub-clinical samples^25,26,53,85^, and have implications for prominent theories of the role of metacognition in depression. Whereas the negativity hypothesis^86^ posits that depressed individuals evaluate themselves in an overly negative way, the depressive realism hypothesis^87^ posits that depressed individuals are more accurate in their evaluations of themselves and that it is non-depressed individuals whose evaluations are distorted by a positivity bias. Under these theories, we would expect depressive symptoms to be associated with either an increase in confidence criteria (negativity hypothesis) or an increase in metacognitive sensitivity/insight (depressive realism). Our results were more in line with the former, as we found no evidence for a relationship between AD symptoms and metacognitive efficiency. Hence, while individuals reporting high levels of AD were more negative in their confidence ratings overall (in line with the negativity hypothesis), this was not associated with a reliable alteration in their ability to dissociate correct from incorrect responses. Indeed, other recent studies have also found no relationship between metacognitive efficiency and anxious-depressive symptoms^28,53^.

The computations underlying metacognitive sensitivity and bias have been suggested to arise from dissociable neural networks. For instance, in the prefrontal cortex (PFC), metacognitive sensitivity is associated primarily with anterior (aPFC) structure and activity^20,88,89^, whereas absolute confidence is associated with ventromedial (vmPFC), posterior medial (mPFC) and dorsolateral (dlPFC) regions^30,90,91^. Our results suggest that anxious-depressive symptoms may be associated with changes in networks subserving absolute confidence, but not metacognitive sensitivity. Intriguingly, recent evidence suggests that interactions between confidence and reward valuation/motivation are reflected in activity in the vmPFC and dorsal anterior cingulate cortex (dACC)^25^. These regions have also been associated with symptoms of apathy^92^, anxiety^93^ and depression^94^ and hence represent promising candidates for the neural locus of the AD effects.

The functional consequences of confidence biases in both AD and CIT should be investigated further. Negative confidence bias may have a pernicious long-term influence on motivation^95,96^, learning^97,98^, information seeking^6^ and self-esteem^53,99^ which in turn may cause and/or exacerbate anxious-depressive symptoms. Conversely, inflated confidence may result in rigid beliefs and cognitive inflexibility, symptoms often observed in OCD^100^, addiction^101^ and schizophrenia^102,103^. Changes in confidence calibration may be linked to maladaptive beliefs about self-efficacy and the level of control one has over their thoughts and/or behaviours. It would be of interest to assess whether successfully challenging these maladaptive beliefs, through techniques such as cognitive behavioural^86^ or metacognitive^104^ therapies, would result in corresponding changes in confidence criteria. As well as providing a useful neuro-computational outcome measure for clinical research^105^, this would help to elucidate a key open question of the causal direction of the relationship between symptoms and metacognitive bias: Do the biases arise prior to, and potentially confer risk for, the onset of symptomology; or are they rather concomitant symptoms themselves? Incorporating quantitative measurement of metacognitive bias into studies employing longitudinal and/or interventional designs could shed light on this question.

We found no evidence that personality traits play a role in the relationships between metacognition and psychopathology. Metacognitive bias related to dimensions of psychopathology directly rather than through a shared link with general psychological dispositions. Indeed, Big-5 dimensions did not predict confidence in either cognitive domain. Interestingly, 1st-order accuracy was negatively associated with extraversion for both tasks. These relationships occurred independently of the accuracy-CIT relationships and, though they were not hypothesized, are in line with previous studies^32,106,107^. Hence, both personality and symptom dimensions were related to 1st-order performance. To elucidate the source of these relationships, future studies may investigate whether factors known to influence decision-making performance, such as choice history bias^108,109^, attention deficits^110^, confirmation bias^111^, and/or alteration in reward/loss sensitivity^81,112^, contribute to the observed 1st-order CIT and/or personality effects. We did not measure IQ here and so it is possible that variation in general intelligence may contribute to the effects, though evidence for relationships between IQ and both extraversion^113,114^ and compulsivity^26,46^ is mixed. Future studies may also investigate whether IQ and/or the recently proposed *d* factor^46^ play a role in the observed 1st-order effects.

Although they were not significantly related to metacognition, personality dimensions were strongly correlated with psychopathology. Numerous relationships with classic diagnostic categories were observed for each Big-5 dimension^34–40^. However, relationships between personality and transdiagnostic symptom dimensions were also found which were masked by the classical categories: positive relationships between SW and conscientiousness, and between CIT and openness. These findings suggest links between personality traits and symptoms which do not neatly fit established diagnostic boundaries, thereby further validating interest in the identification of transdiagnostic symptom predictors^72,73^. Given that the Big-5 represent one level within a hierarchy of traits^115,116^, it would be interesting to investigate exactly which subordinate facets of each dimension are most strongly linked to transdiagnostic symptoms.

Our results have implications for current models of metacognition. A normative model posits that confidence computations reflect the probability of being correct in a statistically optimal manner^117–119^. However, the relationships between symptoms and confidence ratings, and the dissociations between ***d'*** and ***meta-d'*** observed across both tasks, show that the normative model alone cannot fully account for subjective confidence. Rather, our results align with models positing that confidence judgements arise from processes which are dissociable from the decision itself^74,120^.

Both domain-specific and domain-general factors influenced metacognitive performance. At the group-level, objective accuracy was lower for knowledge than perception, but overall metacognitive efficiency and absolute confidence levels were higher. The differences in metacognitive efficiency and confidence criteria between the tasks support an influence of domain-specific factors^30,121^, though it is difficult to identify exactly which as these measures are not only influenced by differences in metacognitive mechanisms between cognitive domains, but also by differences in task characteristics such as 1st-order difficulty^41^ and variability in difficulty across stimulus levels^122^ which were not equalised between tasks. However, a possible explanation for increased metacognitive efficiency in the knowledge task is that, whereas self-evaluation of perceptual task performance required assessment of evidence presented very briefly and then fading in iconic memory, the internally generated knowledge evidence was presumably available to the same degree throughout the trial, including during confidence judgements. Alternatively, given that confidence levels were also higher for the knowledge task here, the increased metacognitive efficiency scores may be explained by a recently discovered positive correlation between efficiency and confidence^1,123^.

In support of domain-general processes also influencing performance, we found significant between-task correlations. In line with previous studies, type-1 accuracy^46^, metacognitive sensitivity^124^ and metacognitive efficiency^125,126^ were all somewhat correlated across tasks. However, overall confidence bias was the most strongly correlated measure^78,79^ and most strongly linked to symptoms. This suggests that a trait-like, global metacognitive process^9,28^ links to psychopathology, as opposed to more ‘local’, domain-specific processes such as uncertainty about sensory evidence or model-based task representations. Global metacognitive evaluations may be intimately linked to beliefs about overall self-efficacy and are likely to have a more pervasive influence on everyday functioning^9,28^. One important consideration is that the task measures of interest here may be affected by different levels of noise^121,127^ and this may have influenced both estimates of their reliability across tasks and the strength of their relationships with other variables (such as symptom scores). For instance, it is possible that estimates of confidence bias may be inherently less noisy than estimates of metacognitive sensitivity and efficiency. Although Meta-d’ measures of metacognitive performance are widely adopted and currently represent the state-of-the-art in the field^41,42,57^, alternative approaches to modelling/quantifying metacognitive abilities^128–130^ are emerging which may be applied in future research to further characterise relationships between metacognition and psychopathology.

Testing symptom variation in the general population affords the advantage of efficient collection of large samples and overcomes the arbitrary boundaries between psychopathology and normality imposed by diagnostic manuals including the DSM^131^ and ICD^132^. However, it remains to be seen whether these results can be extended to clinical samples with the highest levels of symptom severity. The transdiagnostic approach revealed relationships between psychopathology and both metacognition and personality traits which were not apparent in analyses using classic diagnostic categories (see also Rouault et al., 2018^26^), and this may be due to relationships being masked by overlap of symptom dimensions within single categorical disorders, such as overlap of AD and CIT within OCD^9,24,25,47^. This creates challenges both in terms of relating results to previous research and for translation to clinical practice^72^. Future research should investigate whether diagnostic categories (such as OCD) or transdiagnostic dimensions (such as compulsivity) are stronger predictors of cognitive and/or metacognitive deficits in clinical samples. Along these lines, Gillan et al., (2020)^133^ showed that the CIT dimension was a significant predictor of deficits in goal-directed planning whereas having a diagnosis of OCD was not. Furthermore, identification and quantification of relationships between symptoms and cognition at the level of the individual, rather than at the population level^134^, could remain agnostic to over-arching diagnostic labels and provide direct targets for therapeutic intervention, in line with a move towards precision psychiatry^135,136^.

We employed the same battery of questionnaires as previous studies^26,47^ and were able to replicate three previously reported symptom dimensions (AD, CIT, and SW). However, the questionnaire items contributing to these dimensions do not exhaustively cover all forms of psychopathology and other transdiagnostic symptom structures have been proposed^36,51,137,138^ which may capture a broader range of cognitive/metacognitive alterations. It is also important to note that the age ranges of both samples here were heavily skewed towards young adults (Supplementary Figs. S1 and S4), likely due to the online recruitment strategy. Future studies should investigate decision-making and metacognition over extended symptom and age ranges and across different transdiagnostic structures. Additionally, it will be important to ascertain whether relationships between psychopathology and both 1st and 2nd-order decision-making are relatively invariant, or whether they depend on time and context^139^. For instance, the relationships may fluctuate as a function of disorder trajectory or symptom provocation. Understanding temporal dynamics and contextual triggers will help to refine models of the neurocomputational signatures associated with psychopathology and potentially facilitate the identification of novel treatment techniques.

### Supplementary information

Supplementary information

## Data Availability

All data are openly available on the Open Science Framework (OSF) under the URL: https://osf.io/s3cth/.

## Code availability

All code to reproduce the analyses are available on the OSF under the URL: https://osf.io/s3cth/.
