# Supplementary material for: Psychiatrically relevant signatures of domain-general decision-making and metacognition in the general population
Source: Npj Ment Health Res. 2022 Aug 30;1:10. doi: 10.1038/s44184-022-00009-4 (PMC10956036; doi:10.1038/s44184-022-00009-4)
Supplement: Supplementary file 1 — Supplementary information [file 44184_2022_9_MOESM1_ESM.pdf]

Supplementary Information

Psychiatrically relevant signatures of domain-general decision-making and metacognition in the general population

Christopher S.Y. Benwell[1], Greta Mohr[2], Jana Wallberg[1], Aya Kouadio[1], Robin A. A. Ince[2]

[1] Division of Psychology, School of Humanities, Social Sciences and Law, University of Dundee, Dundee, UK

[2] School of Psychology and Neuroscience, University of Glasgow, Glasgow, UK

Supplementary Figures

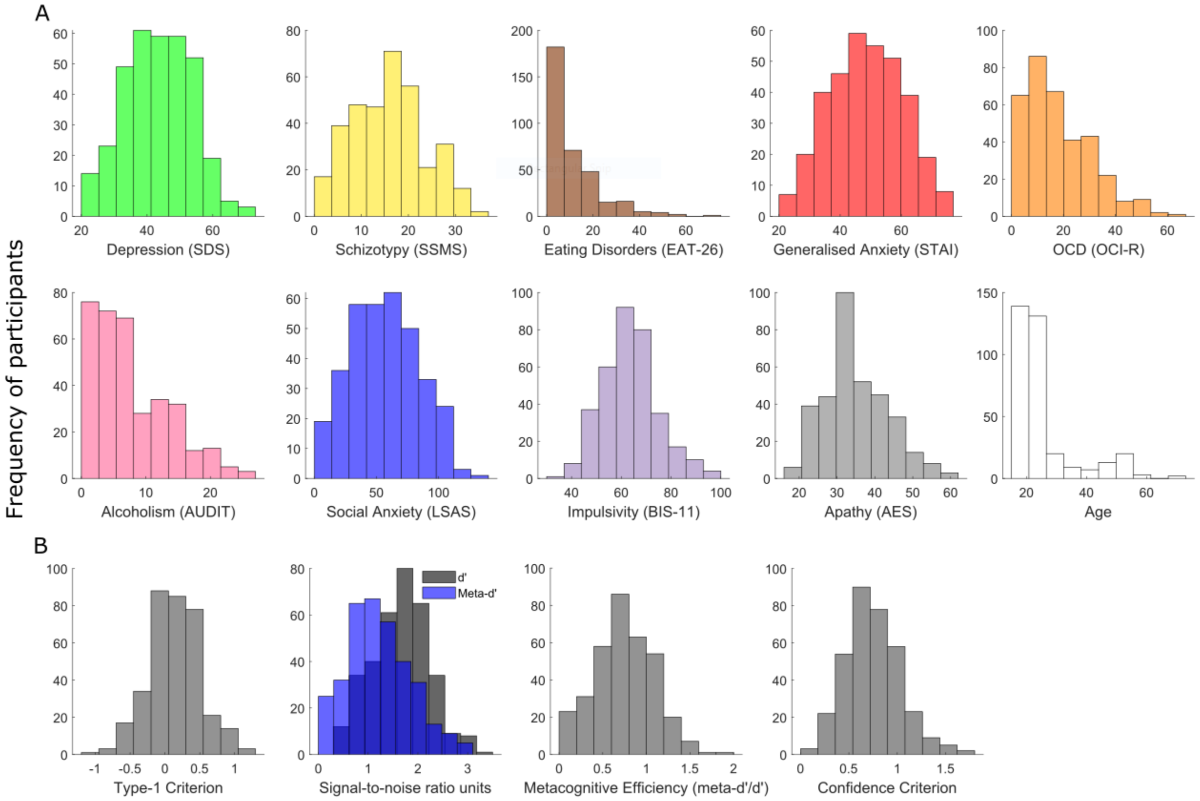

Supplementary Figure 1. Experiment 1 data**.** Distributions of **(A)** psychiatric questionnaire scores, ages, and **(B)** overall Meta-d' task performance measures (collapsed across all levels of perceptual evidence) from experiment 1 (N = 344).

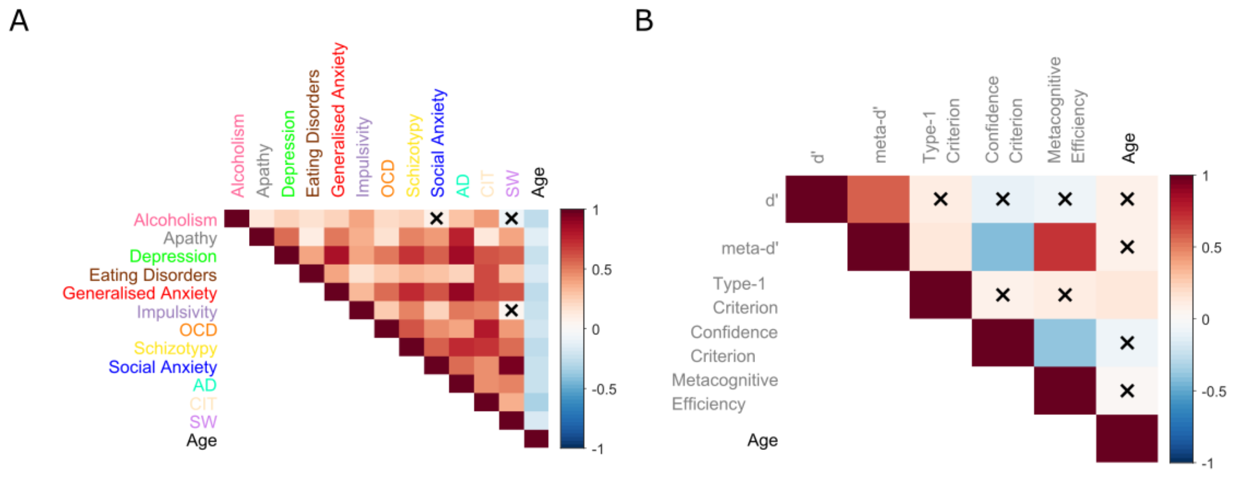

Supplementary Figure 2. Experiment 1 correlations across measures**. (A)** Correlation matrix of psychiatric questionnaire scores, symptom dimension scores and age in experiment 1. **(B)** Correlation matrix of Meta-d' task performance measures and age in experiment 1. X denotes relationship was not statistically significant (p > .05).

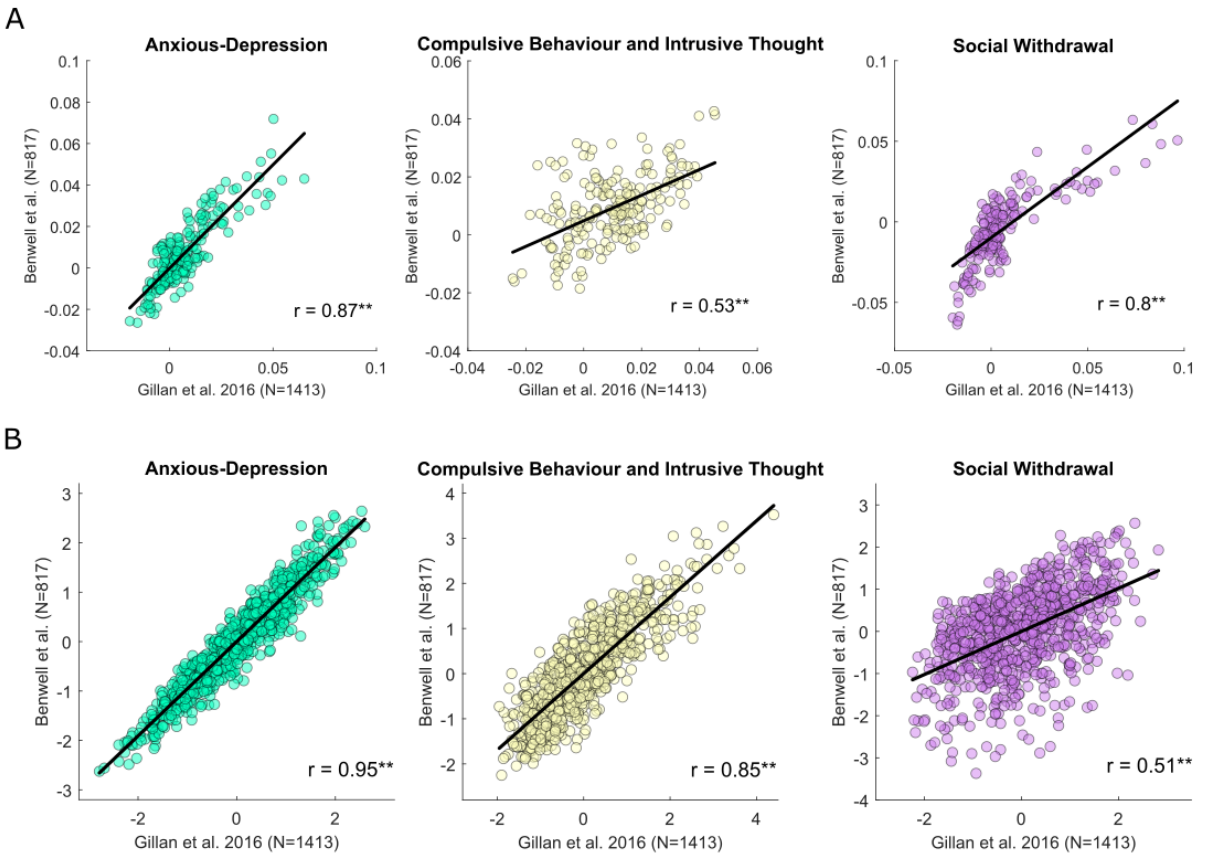

Supplementary Figure 3. Comparison of factor analysis results with Gillan et al., (2016). **(A)** Correlations between questionnaire item weights obtained from the factor analyses in Gillan et al., (2016) and the current study for each of the three psychiatric symptom dimensions. **(B)** Correlations between individual participant factor scores obtained from the factor analyses in Gillan et al., (2016) and the current study for each psychiatric symptom dimension. $**P < 0.001$.

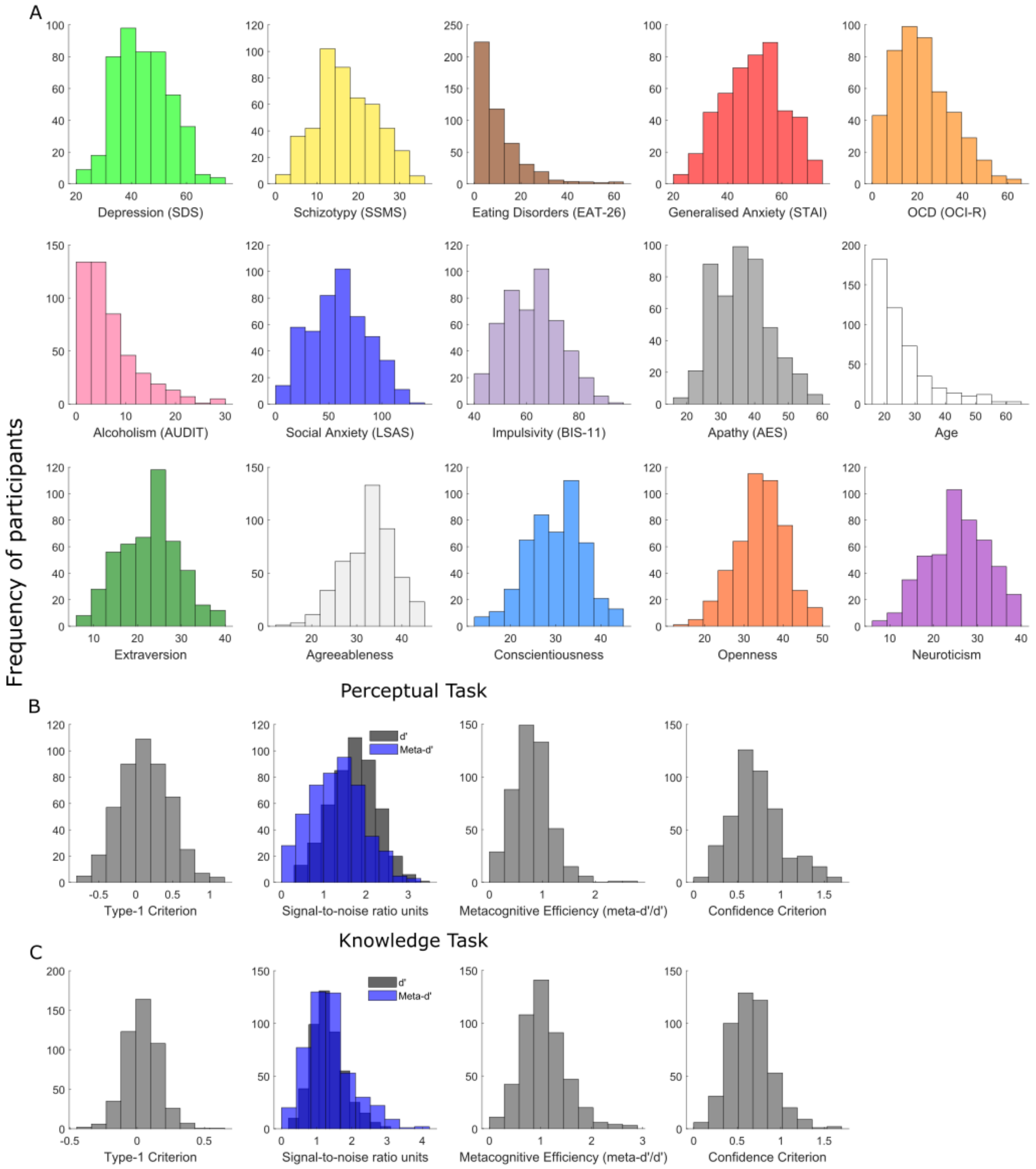

Supplementary Figure 4. Experiment 2 data. Distributions of **(A)** psychiatric questionnaire scores, personality dimension scores, ages, **(B)** overall Meta-d' perceptual task performance measures (collapsed across all levels of evidence), and **(C)** overall Meta-d' knowledge task performance measures from experiment 2 (N = 473).

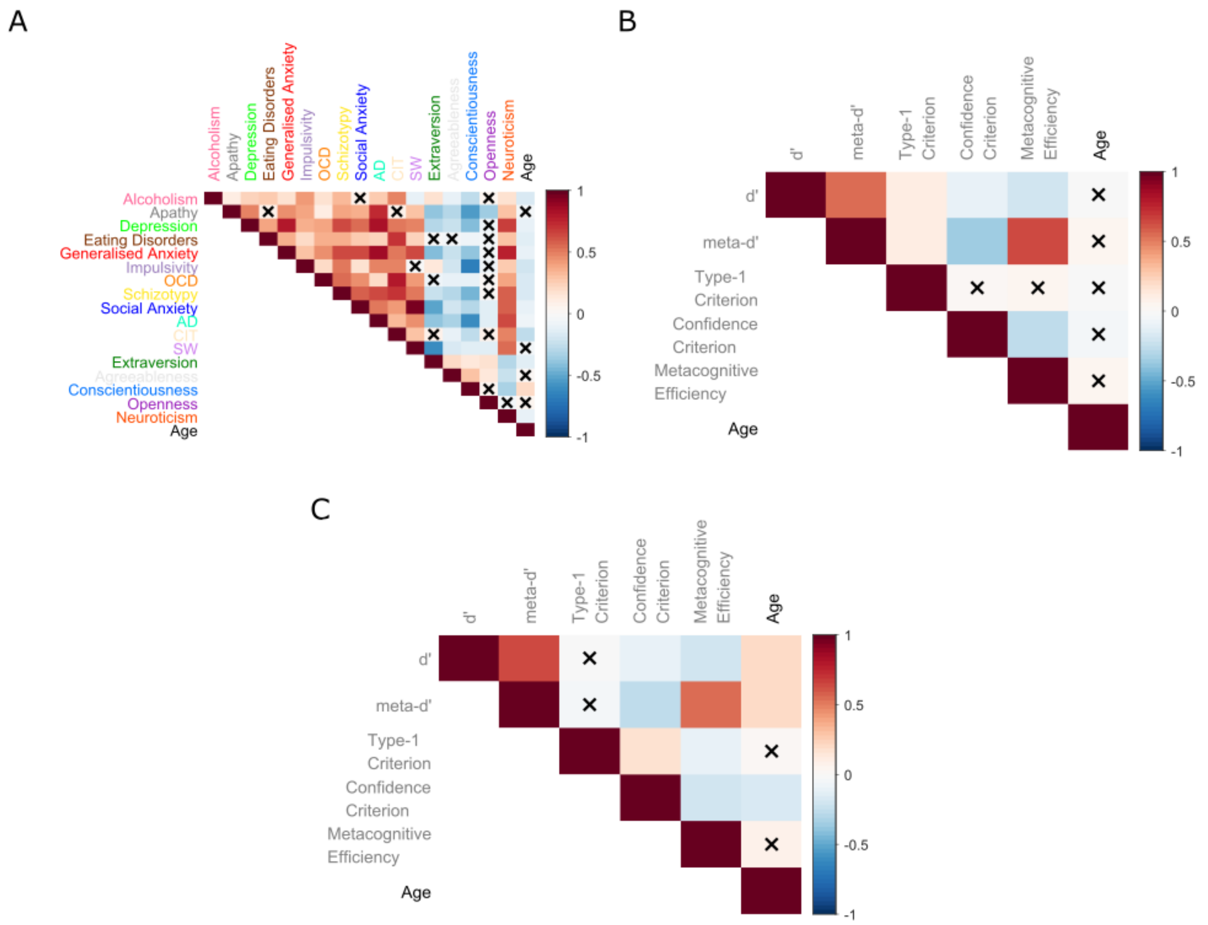

Supplementary Figure 5. Experiment 2 correlations across measures. **(A)** Correlation matrix of psychiatric questionnaire scores, symptom dimension scores, personality dimension scores and age in experiment 2. **(B)** Correlation matrix of Meta-d' perceptual task performance measures and age in experiment 2. **(C)** Correlation matrix of Meta-d' knowledge task performance measures and age in experiment 2. X denotes relationship was not statistically significant (p > .05).

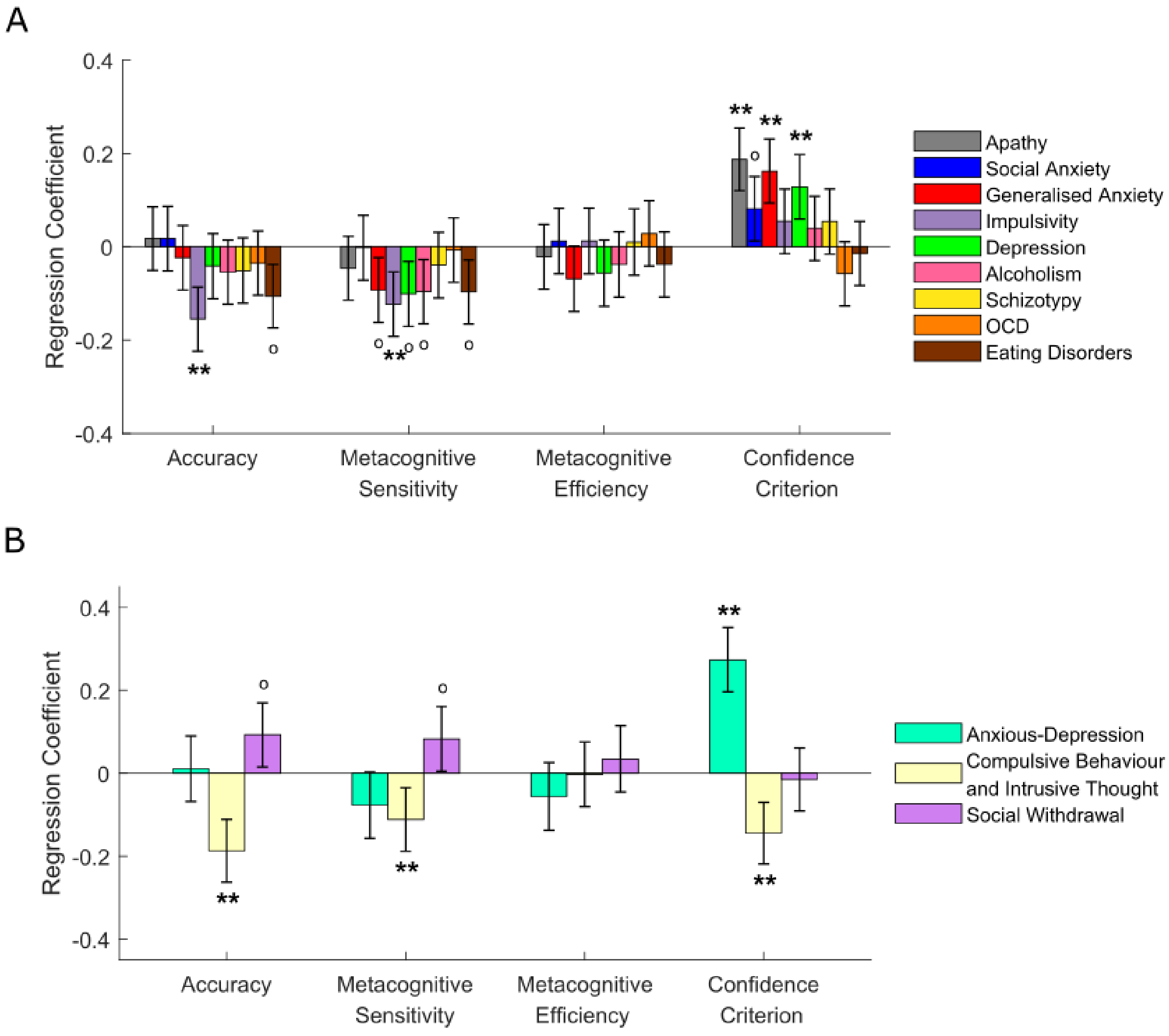

Supplementary Figure 6. Associations between 1st- and 2nd-order decision parameters and self-reported psychopathology, additionally controlling for the influence of age and gender, across both experiments combined (N=817). **(A)** Associations between psychiatric symptom questionnaire scores and Meta-d' parameters from separate regression models. Given that all variables were z-scored prior to entry into the regression models, the y axis indicates the change in each decision parameter (in standard deviations) for each change of 1 standard deviation of questionnaire scores. Accuracy = ***d'***, Metacognitive Sensitivity = ***meta-d'***, Metacognitive Efficiency = log(***meta-d'/d'***). **(B)** Associations between the transdiagnostic symptom dimension scores and Meta-d' parameters from multiple regression models. CIT showed negative relationships with 1st order accuracy, metacognitive sensitivity, and confidence criteria, whereas AD showed only a positive relationship with confidence criteria. No other effects survived multiple comparison correction. All error bars denote 95% Confidence Intervals for the regression coefficients. °P < 0.05 uncorrected; **P < 0.05 corrected for multiple comparisons over the number of dependent variables tested.

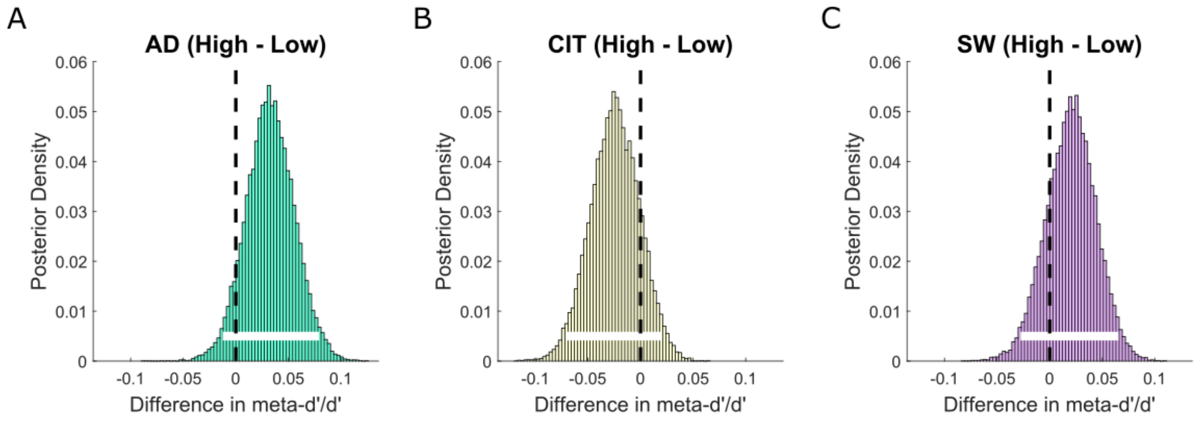

Supplementary Figure 7. Comparing differences in group-level perceptual metacognitive efficiency (***meta-d'/d'***) estimates between high and low symptom groups (created using a median split). Results are shown for the **(A)** anxious-depression (AD), **(B)** compulsive behaviour and intrusive thought (CIT) and **(C)** social withdrawal (SW) dimensions (N = 817). Each histogram represents posterior densities of the difference in mean *meta-d'/d'* between the high and low symptom groups. The white bars indicate the 95% highest-density interval (HDI) for the mean difference in ***meta-d'/d'*** between the groups. For all three symptom dimensions, the 95% HDI includes 0 (vertical dashed line) which indicates that there was no statistically significant difference in metacognitive efficiency between the high and low symptom groups for any dimension.

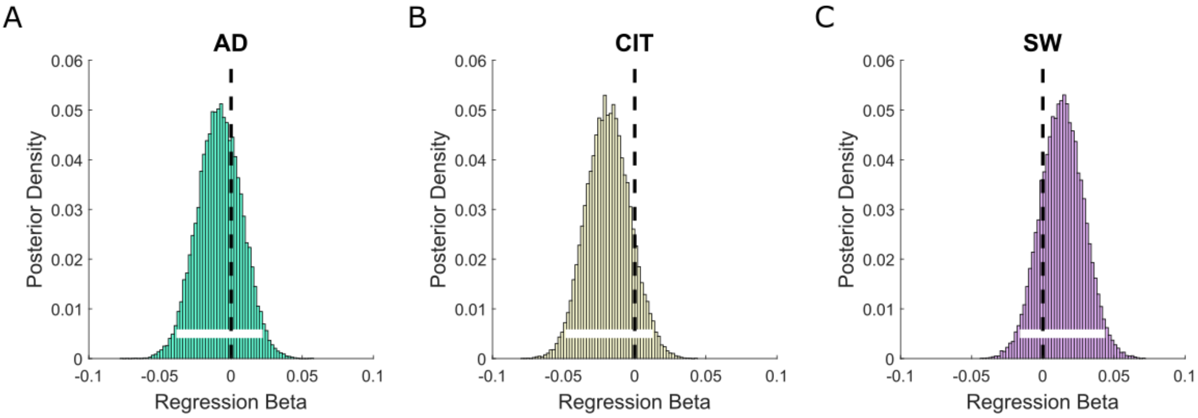

Supplementary Figure 8. Relationships between symptom dimension scores and perceptual metacognitive efficiency (***meta-d'/d'***) estimated within a hierarchical regression analysis. Results are shown for the **(A)** anxious-depression (AD), **(B)** compulsive behaviour and intrusive thought (CIT) and **(C)** social withdrawal (SW) dimensions (N = 817). The regression model was fit using an extension of the HMeta-d model (RHMeta-d) in which the beta regression coefficients were fit simultaneously together with the log(***meta-d'/d'***) scores. Each histogram represents posterior densities of the symptom-metacognitive efficiency regression coefficients. The white bars indicate the 95% highest-density interval (HDI) for the coefficient. For all three symptom dimensions, the 95% HDI includes 0 (vertical dashed line) which indicates that none of the relationships were statistically significant.

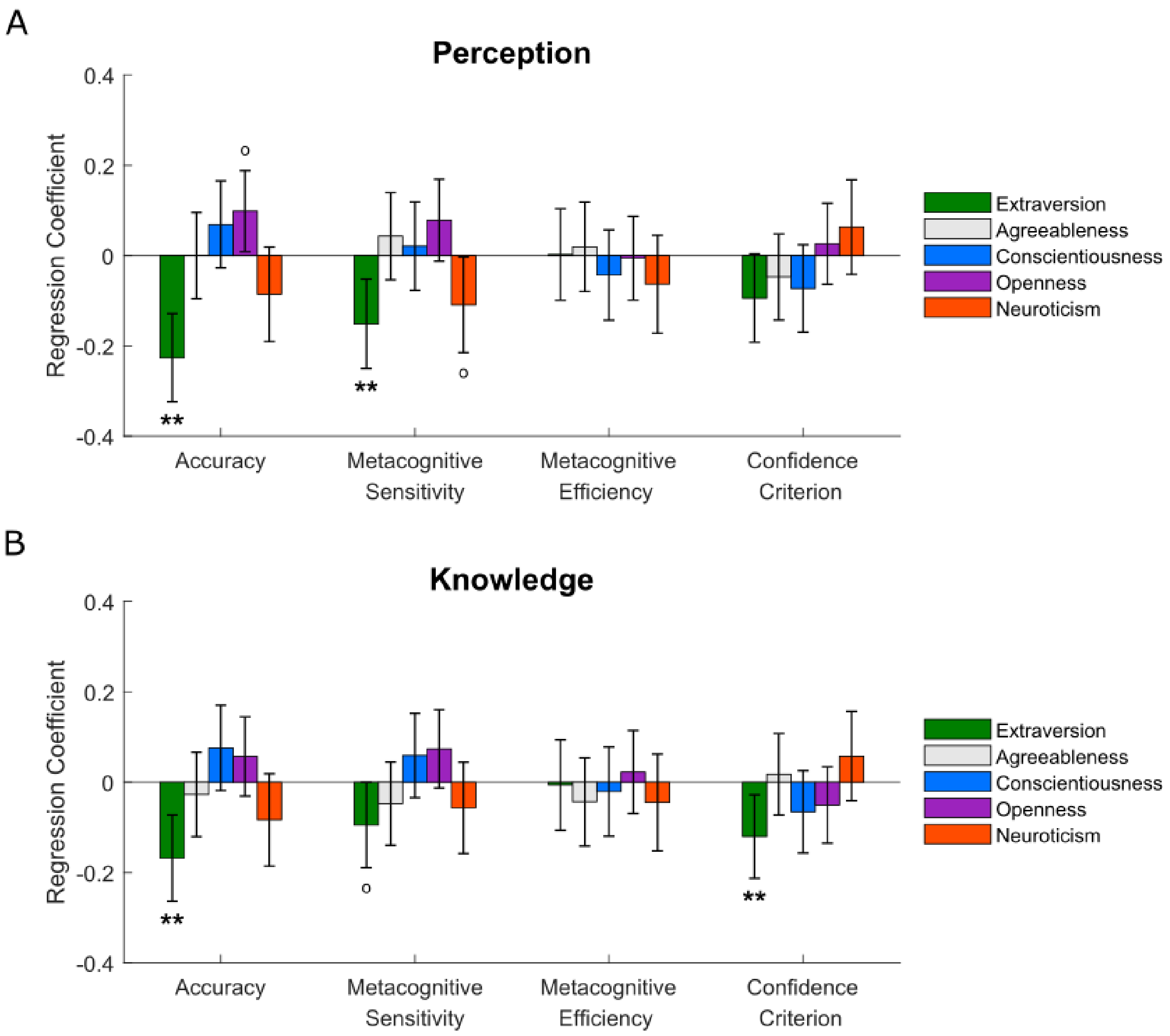

Supplementary Figure 9. Associations between 1st- and 2nd-order decision parameters and self-reported personality traits, controlling for age and gender, in experiment 2. **(A)** Associations between personality dimension scores and perception Meta-d' parameters. The y axis indicates the change in each decision parameter for each change of 1 standard deviation of personality dimension scores. Accuracy = ***d'***, Metacognitive Sensitivity = ***meta-d'***, Metacognitive Efficiency = log(***meta-d'/d'***). **(B)** Associations between personality dimension scores and knowledge Meta-d' parameters. All error bars denote 95% Confidence Intervals for the regression coefficients. °P < 0.05 uncorrected; **P < 0.05 corrected for multiple comparisons over the number of dependent variables tested.

Supplementary Results

Relationships between self-reported psychiatric symptoms, task measures and both age and gender in study 1

In line with previous research[1], we found that older age was associated with lower levels of self-reported psychopathology across all 9 questionnaires (all *Pearson r's* $\leq$ -0.15, all *p's* $\leq$ .004) (Supplementary Figure 2A). However, age was not significantly associated with any of the task measures of interest (Supplementary Figure 2B). Female participants reported higher symptoms of depression ($t(342) = 2.93$, $p = .004$), eating disorders ($t(342) = 3.45$, $p < .001$), generalised anxiety ($t(342) = 3.06$, $p = .002$) and social anxiety ($t(342) = 3.57$, $p < .001$), but lower symptoms of apathy ($t(342) = -2.17$, $p = .03$), relative to male participants. Additionally, female participants showed lower perceptual ($t(342) = -3.57$, $p < .001$) and metacognitive ($t(342) = -1.98$, $p = .048$) sensitivity relative to males, along with higher confidence criteria (thereby indicating lower levels of absolute confidence) ($t(342) = 2.16$, $p = .032$).

Relationships between self-reported psychiatric symptoms, task measures and both age and gender in study 2

Similar relationships were found between both age and gender and the symptom questionnaire scores and perceptual task measures as those observed in experiment 1 (see Supplementary Figure 5). In terms of the personality dimensions, age was negatively associated with both extraversion ($r(471) = -0.15$, $p = .001$) and neuroticism ($r(471) = -0.12$, $p = .01$), but positively associated with conscientiousness ($r(471) = 0.2$, $p < .001$) (Supplementary Figure 5A). Female participants reported higher levels of extraversion, agreeableness, and neuroticism (all *t*'s $\geq$ 2.40, all *p*'s $\leq$ .017) relative to male participants. In contrast to the perceptual task measures where no age-related effects were observed (Supplementary Figure 5B), age was positively associated with both knowledge *d'* ($r(471) = 0.22$, $p < .001$) and *meta-d'* ($r(471) = 0.21$, $p < .001$) scores (indicating increased accuracy and metacognitive sensitivity with age) and negatively associated with confidence criteria (thereby

indicating increased levels of absolute confidence with age) ($r(471) = -0.16$, $p < .001$) (Supplementary Figure 5C). In line with the perceptual task, female participants showed lower 1st-order accuracy (*d'*) ($t(471) = -4.78$, $p < .001$) and metacognitive sensitivity (*meta-d'*) ($t(471) = -6.76$, $p < .001$) on the knowledge task relative to males, along with higher confidence criteria (thereby indicating lower levels of absolute confidence) ($t(471) = 8.06$, $p < .001$).

Further analyses investigating the relationship between symptom dimensions and metacognitive efficiency

We directly compared group-level estimates of metacognitive efficiency (***meta-d'/d'***) for the perception task (data collapsed across both experiments: N = 817) between 'high' and 'low' symptom groups (created using a median split) across all three symptom dimensions using the hierarchical Bayesian fitting routine in the 'HMeta-d'' toolbox[2]. The results are presented in Supplementary Figure 7. No significant differences in metacognitive efficiency were found between high and low symptom groups for AD (High AD mean = 0.87, low AD mean = 0.84, 95% highest density interval (HDI) for difference in means between groups = [-0.01 0.08]), CIT (High CIT mean = 0.84, low CIT mean = 0.86, 95% HDI for difference in means between groups = [-0.07 0.02]), or SW (High SW mean = 0.86, low SW mean = 0.84, 95% HDI for difference in means between groups = [-0.03 0.06]).We also tested for group differences separately at each of the 8 individual difficulty levels to test whether symptoms might relate to changes in metacognitive efficiency at certain levels of task difficulty. However, no significant differences in metacognitive efficiency between groups were found at any difficulty level for any of the three dimensions (All 95% HDI's included 0).

In a final test for potential relationships between symptom dimensions and metacognitive efficiency, we estimated their strength within a hierarchical meta-d' regression model (RHMeta-d)[2,3]. The regressors included in the hierarchical model were Age, Gender, AD scores, CIT scores and SW scores, with the outcome variable being metacognitive efficiency (***meta-d'/d'***) scores. The results are presented in Supplementary Figure 8. None of the relationships were statistically significant, with all

95% HDI's for the regression coefficients including 0 (AD β = -0.008, 95% HDI = [-0.04 0.02]; CIT β = -0.019, 95% HDI = [-0.05 0.01]; SW β = 0.014, 95% HDI = [-0.016 0.044]). Hence, we found no evidence across any of our analyses for a relationship between psychiatric symptoms and metacognitive efficiency.

Relationships between Big-5 Factors and task measures without accounting for symptom dimensions

We constructed regression models with Big-5 scores (see Supplementary Figure 4A for full sample distributions) entered simultaneously (along with age and gender) as predictors of each outcome measure separately for each task. For the perceptual task (Supplementary Figure 9A), self-reported extraversion was associated with reduced perceptual accuracy ($\beta = -0.23$, $p < .001$, corrected) and metacognitive sensitivity ($\beta = -0.15$, $p = .015$, corrected). Trait openness was associated with increased perceptual accuracy ($\beta = 0.10$, $p = .032$, uncorrected) and neuroticism was associated with reduced metacognitive sensitivity ($\beta = -0.11$, $p = .044$, uncorrected), but neither of these relationships survived correction. No personality dimensions were significantly associated with either metacognitive efficiency or confidence criteria for the perceptual task (all *p*'s ≥ .05). For the knowledge task (Supplementary Figure 9B), only extraversion was significantly associated with any task measures, predicting reduced 1st-order accuracy ($\beta = -0.17$, $p < .001$, corrected), reduced metacognitive sensitivity ($\beta = -0.10$, $p = .049$, uncorrected) and reduced confidence criteria (indicating high levels of absolute confidence) ($\beta = -0.12$, $p = .044$, corrected).

Supplementary References

1. Rouault, M., Seow, T., Gillan, C. M. & Fleming, S. M. Psychiatric Symptom Dimensions Are Associated With Dissociable Shifts in Metacognition but Not Task Performance. *Biol. Psychiatry* **84**, 443–451 (2018).
2. Fleming, S. M. HMeta-d: hierarchical Bayesian estimation of metacognitive efficiency from confidence ratings. *Neurosci. Conscious.* **2017**, (2017).
3. Harrison, O. K. *et al.* The Filter Detection Task for measurement of breathing-related interoception and metacognition. *Biol. Psychol.* **165**, 108185 (2021).
